# Corticosteroid use in COVID-19 patients: a systematic review and meta-analysis on clinical outcomes

**DOI:** 10.1186/s13054-020-03400-9

**Published:** 2020-12-14

**Authors:** Judith van Paassen, Jeroen S. Vos, Eva M. Hoekstra, Katinka M. I. Neumann, Pauline C. Boot, Sesmu M. Arbous

**Affiliations:** 1 Department of Intensive Care, Leiden University Medical Center, Albinusdreef 2, 2333 ZA Leiden, The Netherlands; 2 Faculty of Medicine, Leiden University Medical Center, Leiden, The Netherlands; 3 Department of Clinical Epidemiology, Leiden University Medical Center, Leiden, The Netherlands

**Keywords:** COVID-19, SARS-CoV-2, Coronavirus, Corticosteroids, Mortality, Viral clearance, Mechanical ventilation

## Abstract

**Background:**

In the current SARS-CoV-2 pandemic, there has been worldwide debate on the use of corticosteroids in COVID-19. In the recent RECOVERY trial, evaluating the effect of dexamethasone, a reduced 28-day mortality in patients requiring oxygen therapy or mechanical ventilation was shown. Their results have led to considering amendments in guidelines or actually already recommending corticosteroids in COVID-19. However, the effectiveness and safety of corticosteroids still remain uncertain, and reliable data to further shed light on the benefit and harm are needed.

**Objectives:**

The aim of this systematic review and meta-analysis was to evaluate the effectiveness and safety of corticosteroids in COVID-19.

**Methods:**

A systematic literature search of RCTS and observational studies on adult patients was performed across Medline/PubMed, Embase and Web of Science from December 1, 2019, until October 1, 2020, according to the PRISMA guidelines. Primary outcomes were short-term mortality and viral clearance (based on RT-PCR in respiratory specimens). Secondary outcomes were: need for mechanical ventilation, need for other oxygen therapy, length of hospital stay and secondary infections.

**Results:**

Forty-four studies were included, covering 20.197 patients. In twenty-two studies, the effect of corticosteroid use on mortality was quantified. The overall pooled estimate (observational studies and RCTs) showed a significant reduced mortality in the corticosteroid group (OR 0.72 (95%CI 0.57–0.87). Furthermore, viral clearance time ranged from 10 to 29 days in the corticosteroid group and from 8 to 24 days in the standard of care group. Fourteen studies reported a positive effect of corticosteroids on need for and duration of mechanical ventilation. A trend toward more infections and antibiotic use was present.

**Conclusions:**

Our findings from both observational studies and RCTs confirm a beneficial effect of corticosteroids on short-term mortality and a reduction in need for mechanical ventilation. And although data in the studies were too sparse to draw any firm conclusions, there might be a signal of delayed viral clearance and an increase in secondary infections.

## Background

Since the start of the outbreak, Coronavirus disease 2019 (COVID-19), caused by the novel coronavirus SARS-CoV-2, has spread globally from Wuhan, China. A total of 40,559,736 cases have been reported, and 1,121,499 people have died as of October 19. [1] Many countries have been affected, causing immense stress on healthcare systems worldwide. This is the third epidemic caused by a coronavirus, after severe acute respiratory syndrome (SARS) in 2002 and Middle East respiratory syndrome (MERS) in 2012 [2, 3]. The clinical presentation ranges from asymptomatic or mild disease to severe pneumonia in which the most severe cases deteriorate with acute respiratory distress syndrome (ARDS) requiring prolonged mechanical ventilation, or even extracorporeal membrane oxygenation (ECMO) [4, 5]. Approximately 16–35% develop severe pneumonia, 2–17% need mechanical ventilation, of whom up to 15% need ECMO therapy, [6–8] and the case fatality rate is 1.4–15% [5, 9, 10]. In the pathophysiology of severe COVID-19, the host immune response plays a key role and it has become evident that COVID-19 pneumonia is associated with both hyper inflammation and immunoparalysis [11]. A clinical presentation of massive vascular inflammation, disseminated coagulation, shock and ARDS is frequently triggered [9–11].

Though many therapies aiming at mitigation of the inflammatory response are being evaluated, strong evidence of benefit is lacking. Corticosteroids might have beneficial effects in overcoming both hyperinflammation and ARDS [4, 15–17]. Furthermore, they could serve as an easily accessible and affordable treatment option. On the other hand, there are known adverse effects of corticosteroid use, such as delayed viral clearance, opportunistic infections and suppression of the hypothalamic-pituitary-adrenal axis [2, 18, 19]. Earlier studies done in MERS-CoV and SARS-CoV showed delayed viral clearance, opportunistic infections and hyperglycemia [20–22]. Therefore, a high number of observational studies and randomized controlled trials (RCT) on corticosteroids for COVID-19 have been initiated and reported, and the signal is a beneficial effect. The RECOVERY trial was the first to report that the use of dexamethasone as opposed to usual care reduced 28-day mortality in patients requiring oxygen therapy or mechanical ventilation [23]. And a prospective meta-analysis of seven randomized clinical trials showed that administration of corticosteroids was associated with lower 28-day all-cause mortality [24]. And while initially the World Health Organization (WHO) recommended against corticosteroid treatment, as of September 2, 2020, the WHO recommends systemic corticosteroids rather than no systemic corticosteroids for the treatment of patients with severe and critical COVID-19 [15, 25]. Also, the Surviving Sepsis Guideline on management of COVID-19 recommends administration of steroids in patients with severe COVID-19 on mechanical ventilation with ARDS and in patients with COVID-19 and refractory shock [26].

However, the effectiveness and safety of corticosteroids still remain uncertain, because of scarcity of RCTs and inconclusive observational studies, and reliable data to further shed light on the benefit and harm are needed. Therefore, the aim of this systematic review and meta-analysis of observational studies and RCTs was to evaluate the effectiveness and safety of corticosteroids in COVID-19.

## Methods

### Data sources and search strategy

A systematic review according to the PRISMA guidelines was conducted [27]. The meta-analysis was retrospectively registered under number 38752 at ISRCTN.org. A comprehensive systematic search was conducted for published studies in Medline/PubMed, Embase and Web of Science from December 1, 2019, to October 1, 2020. The search strategy consisted of the components “COVID-19,” “intensive care” and “corticosteroids” (Additional file 1).

### Eligibility

RCTs and observational cohort studies assessing the effect of corticosteroids in COVID-19 were eligible if they met the following inclusion criteria: adult patients (age ≥ 18 years), COVID-19 patients diagnosed by reverse transcriptase polymerase chain reaction (RT-PCR), reporting on outcome measures in relation to corticosteroid treatment, corticosteroids not restricted with respect to type, dose and duration. Studies concerning pregnant women or children, reviews, case series including less than 15 patients and articles that were not available in English were excluded [28].

### Definition of primary and secondary outcomes

The primary outcomes were *short-term mortality* (i.e., short-term mortality as defined in the study, including 28-day, 30-day and hospital mortality) and *viral clearance* (i.e., as defined by the study, based on RT-PCR in respiratory specimens). Secondary outcomes were: *mechanical ventilation* (i.e., as defined by the study: need for invasive mechanical ventilation, duration of mechanical ventilation, ventilator-free days or other oxygen therapy), *length of hospital stay* (LOS-hospital) and *secondary infections.* For exact used definitions see Additional file 2.

### Study selection

Suitable studies were selected in two stages. First, six independent reviewers screened all selected titles and abstracts (JvP, JV, EH, KN, PB, SA). If there was consensus that a study was unsuitable for inclusion, it was excluded. Next, the full-text articles were screened independently by two authors and included if both authors agreed. If needed, the article was discussed with the third reviewer until consensus was reached.

### Data extraction and quality analysis

After selection, data were extracted by one and checked by a second investigator (JvP, JV, EH, KN, PB). For each study, the author, journal, country, city and hospital in which the study was conducted, date of start of inclusion, study population, study groups, type, dose, route of administration of corticosteroids, median time before corticosteroid initiation, duration of administration, primary and secondary outcomes and adverse events at any time point after admission were extracted in a standardized data extraction form (Additional file 2).

For each individual study, the quality was assessed. For RCTs, the risk of bias was assessed on six domains (random sequence generation, concealment of allocation, blinding, selective outcome reporting, incomplete outcome data and other) [29, 30]. The Newcastle Ottawa Scale was used for validity assessment of observational studies [31, 32]. The NOS score ranges from 0 (low quality) to 9 (high quality) points.

### Data analysis and reporting

For the effect of corticosteroids on *mortality*, a pooled estimate was calculated and graphically summarized in a forest plot. Data from observational studies were analyzed separately from the RCTs, and both the separate results and the overall combined outcomes were calculated and summarized in the plot. When available, the *adjusted* odds ratio (OR) or relative risk (RR) from the cohort studies were used for pooling to reduce confounding. Since the endpoint (mortality) occurred relative infrequently, the OR will be close to the RR and therefore we decided to pool both RR and OR estimates of the individual studies [33]. Furthermore, a pooled estimate was calculated and graphically summarized in a forest plot for *need for mechanical ventilation*.

To allow studies to have a different underlying effect, a random effects model was used. I^2^ statistics was used to quantify heterogeneity. Furthermore, for the pooled estimate of effect on mortality, tau^2^ was used to assess the variance of the true effects. The GRADE approach was used to assess the quality of the evidence for the effect of corticosteroids on mortality. STATA 16.0 was used to perform data analysis.

## Results

### Study selection

**Fig. 1:**
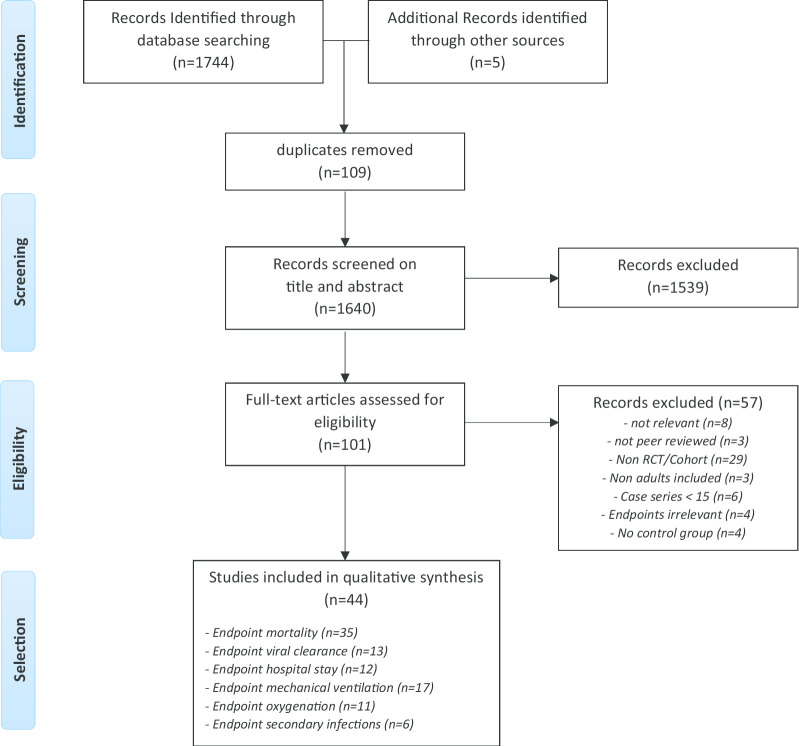
Flowchart article selection.docx

Our search yielded 1640 unique studies. After qualification of title and abstract, 101 studies were selected for full review. Based on exclusion criteria, 57 additional studies were excluded (references in Additional file 3). The remainder of 44 studies, comprising 20.197 patients, was included in this systematic review and meta-analysis. (Fig. 1).

### Study characteristics (Table 1 and Additional file 4)

**Table 1:** Study characteristics

	Author	Reference	Study type	Type—dose ^c^ corticosteroids	Sample size	CoVID—Study population	Reporting outcome^a^						Quality score^δ^ *(Risk of bias or NOS*)	Main findings
							M	V	H	R	O	I		
1	Angus	34	REMAPt ^b^	Hydrocortisone < 1 mg/kg ED	403	ICU patients	x		x	x			Risk of bias^d^	Two hydorcoritsone dosing resulted high probabilities of superiority with regard to the odds of improvement in organ support–free days within 21 days, compared to standard of care
2	Bani-Sadr	39	Cohort with historical controls	Prednisolone or Methylprednisolone ≥ 1 mg/kg ED	319	Hospitalized patients	x				x	x	4	Addition of corticosteroids to our institution’s COVID-19 treatment protocol was associated with a significant reduction in hospital mortality in the “after” period
3	Cao	80	Retrospective Observational	Unknown	102	Hospitalized patients	x						5	Patient characteristics seen more frequently in those who died were development of systemic complications following onset of the illness and the severity of disease requiring admission to the ICU
4	Chen Zu		Retrospective Observational	Unknown	267	Hospitalized patients	x	x					7	Corticosteroid treatment is associated with prolonged viral RNA shedding and should be used with caution
5	Chroboczek	72	Retrospective Observational	Unknown	70	Hospitalized patients				x			6	Corticosteroids therapy affected the risk of intubation with a risk difference of − 47.1% (95% CI − 71.8 to − 22.5)
6	Dequin	35	Randomized controlled trial	Methylprednisolone or hydrocortisone < 1 mg/kg ED	149	ICU patients with respiratory failure	x			x	x	x	Risk of Bias^d^	Low-dose hydrocortisone, compared with placebo, did not significantly reduce treatment failure (defined as death or persistent respiratory support) at day 21 in critically ill patients
7	Fadel	38	Quasi experimental	Methylprednisolone ≥ 1 mg/kg ED	213	Moderate-to-severe CoVID patients	x		x	x			6	An early short course of methylprednisolone in patients with moderate-to-severe COVID-19 reduced escalation of care and improved clinical outcomes
8	Fang Mei	40	Retrospective Observational	Methylprednisolone < 1 mg/kg ED	78	Hospitalized patients		x					5	Low-dose corticosteroid therapy may not delay viral clearance in patients with COVID-19
9	Feng Ling	66	Retrospective Observational	Unknown	476	Hospitalized patients	x		x				5	Differences in AT II receptor inhibitors use were associated with different severities of disease. Multiple lung lobes involvement and pleural effusion were associated with the severity of COVID-19. Advanced age (> 75 yr) was a risk factor for mortality
10	Fernandez	41	Retrospective Observational	Methylprednisolone ≥ 1 mg/kg ED	463	Patients with ARDS hyperinflammation	x				x		5	Glucocorticoid use is associated with increased survival and improved mortality rates in severe CoVID-19 patients
11	Gazzaruso	42	Retrospective Observational	Methylprednisolone or prednisone < 1 mg/kg ED	219	Hospitalized patients	x				x		3	Antirheumatic drugs, probably steroids included, may modulate inflammation and avoid a hyperinflammation that leads to severe complications and death in subjects with COVID-19
12	Gong Guan	43	Retrospective Observational	Methylprednisolone ≥ 1 mg/kg ED	34	Hospitalized Patients < 50 years		x			x		6	Corticosteroids therapy can effectively release COVID‐19 symptoms, improve oxygenation and prevent disease progression. However, it can prolong the negative conversion of nucleic acids
13	Horby	23	Randomized controlled trial	Dexamethasone < 1 mg/kg ED	6425	Hospitalized patients	x		x	x			Risk of bias^d^	The use of dexamethasone resulted in lower 28-day mortality among those who were receiving either invasive mechanical ventilation or oxygen alone at randomization but not among those receiving no respiratory support
15	Hu Wang	44	Retrospective Observational	Prednisolone or methylprednisolone > 1 mg/kg ED	308	Hospitalized patients	x	x					4	Glucocorticoid therapy did not significantly influence the outcomes nor the adverse events of COVID-19 pneumonia
16	Huang Song	45	Retrospective Observational	Methylprednisolone 2 study groups: High: ≥ 1 mg/kg ED Low: < 1 mg/kg ED	64	Hospitalized patients	x						4	There were no significant differences in the duration of severe illness or the number of days on high level respiratory support between low-dose and high-dose methylprednisolone group. The mean number of days in the hospital was higher in the high-dose group
14	Huang Yang	81	Retrospective Observational	Unknown	60	Severe CoVID patients						x	5	There were no statistically significant differences in immunoglobulin therapy and GCs therapy between the improvement and deterioration subgroups
17	Jeronimo	36	Randomized controlled trial	Methylprednisolone < 1 mg/kg ED	393	Hospitalized patients	x		x	x		x	Risk of bias^d^	Results showed no overall reduction in mortality in 28 days. Patients over 60 years presented a lower mortality in a subgroup analysis
18	Keller	73	Retrospective Observational	Unknown	1806	Early hospitalized patients	x			x			6	In high CRP group, glucocorticoids show significantly reduced risk of mortality or mechanical ventilation (odds ratio, 0.23; 95% CI, 0.08–0.70). In low CRP group, glucocorticoids were associated with significantly increased risk of mortality or mechanical ventilation (OR, 2.64; 95% CI,1.39–5.03)
19	Li Hu	46	Retrospective Observational	Methylprednisolone high and low ED	203	Hospitalized patients		x					5	A dose response relation is suggested for corticosteroids on viral shedding. In addition, high-dose but not low-dose corticosteroids were found to potentially increase mortality in severe patients
20	Li Li	47	Retrospective Observational	Methylprednisolone or prednisone < 1 mg/kg ED	475	Non-severe CoVID patients	x	x	x			x	5	Early, low-dose, and short-term corticosteroids therapy was associated with worse clinical outcomes
21	Li Zhou	48	Retrospective Observational	Methylprednisolone > 1 mg/kg ED	187	Radiologically progressive CoVID patients				x	x		6	Short-term, low-to-moderate-dose corticosteroids benefits patients with LDH levels of less than two times the ULN, who may be in the early phase of excessive inflammation
22	Lui Fang	49	Retrospective Observational	Methylprednisolone ≥ 1 mg/kg ED	101	Hospitalized patients	x						3	The majority of patients present primarily with fever and typical manifestations on chest imaging. Middle-aged and elderly patients with underlying comorbidities are susceptible to respiratory failure and may have a poorer prognosis
23	Liu Zhang	81	Retrospective Observational	Unknown	1190	Hospitalized patients	x						5	Treatment with glucocorticoids increased the risk of progression from not severe to severe disease (OR 3.79, 95% CI 2.39–6.01)
24	Liu Zheng	50	Retrospective Observational	Methylprednisolone ≥ 1 mg/kg ED	101	Hospitalized patients		x			x		5	Timely and appropriate application of methylprednisolone in severe and critical patients may improve outcomes and lung function without negative impacts on specific SARS-CoV-2 IgG production
25	Lu Chen	51	Retrospective Observational	Methylprednisolone, hydrocortisone or dexamethasone > 1 mg/kg ED	244	Hospitalized patients	x				x		7	Limited effect of corticosteroid therapy could pose to overall survival of critically ill patients with COVID-19. Given the adverse effects, corticosteroid therapy must be commenced with caution, and prudent dosage should be promoted under certain circumstances
26	Ma Qi	52	Retrospective Observational	Methylprednisolone 2 study groups: High: ≥ 1 mg/kg ED Low: < 1 mg/kg ED	72	Severe and critical patients	x	x	x	x			6	Corticosteroids cannot reduce the hospital mortality and is not associated with delayed viral clearance, but it could relieve the inflammatory storm and improve clinical symptoms in brief. Patients with severe COVID-19 could benefit from low-dose corticosteroids
27	Ma Zeng	53	Retrospective Observational	Methylprednisolone ≥ 1 mg/kg ED	450	Severe and non-severe patients	x	x	x	x		x	4	Corticosteroids use may be accompanied by increased use of antibiotics, longer hospitalization, and prolonged viral shedding
28	Majmundar	54	Retrospective Observational	Prednisolone, dexamethasone, methylprednisolone > 1 mg/kg ED	205	Hospitalized patients	x		x	x	x		6	Corticosteroids were associated with a significantly lower risk of the ICU transfer, intubation, or in-hospital death,
29	Mikulska	55	Retrospective Observational	Methylprednisolone high and low ED	215	Hospitalized non-intubated patients	x				x		6	Early adjunctive treatment with tocilizumab, methylprednisolone or both may improve outcomes in non-intubated patients
30	Nelson	56	Retrospective Observational	Methylprednisolone ≥ 1 mg/kg ED	117	ICU patients on mechanical ventilation	x		x	x			8	Methylprednisolone was associated with increased ventilator-free days and higher probability of extubation in a propensity-score matched cohort
31	Rodriquez	57	Retrospective Observational	Methylprednisolone ≥ 1 mg/kg ED	1014	Hospitalized patients	x			x	x		7	Tocilizumab should be prioritized for being tested in randomized trials targeting patients with data suggestive of a hyperinflammatory state. The results for PDC were less consistent but are also encouraging
32	Rubio	68	Retrospective Observational	Unknown	92	ICU and general ward patients	x			x			5	The early use of GC pulses could reduce the use of tocilizumab and might decrease events such as intubation and death
33	Salton	58	Retrospective Observational	Methylprednisolone ≥ 1 mg/kg ED	173	ARDS patients	x		x	x			8	Per-protocol administration of prolonged low-dose methylpred-nisolone treatment is associated with a significantly lower hazard of death, reduced ICU burden and decreased ventilator dependence
34	Shen Zheng	59	Retrospective Observational	Methylprednisolone unknown dose	325	Hospitalized patients		x					4	COVID-19 cases in Shanghai were imported. Rapid identification and effective control measures helped to contain the outbreak and prevent community transmission
35	Shi Wu	71	Retrospective Observational	Unknown	99	Hospitalized patients		x					4	SARS-CoV-2 RNA clearance time was associated with sex, disease severity and lymphocyte function. The current antiviral protocol and low-to-moderate dosage of corticosteroid had little effect on the duration of viral excretion
36	Tomazini	37	Randomized controlled trial	Dexamethasone > 1 mg/kg ED	299	ICU patients with moderate-to-severe ARDS	x			x		x	Risk of bias^d^	Dexamethasone plus standard care compared with standard care alone resulted in a significant increase in the number of ventilator-free days (days alive and free of mechanical ventilation) over 28 days
37	Wang Jiang	60	Retrospective Observational	Methylprednisolone > 1 mg/kg ED	46	Severe hospitalized patients	x			x	x		7	early, low-dose and short-term application of methylprednisolone was associated with better clinical outcomes in severe CoVID-19 patients and should be considered before onset of ARDS
38	Wang Yang	67	Retrospective Observational	Unknown	69	Hospitalized patients	x						4	COVID-19 shows frequently fever, dry cough, and increase of inflammatory cytokines, and induced a mortality rate of 7.5%. Older patients or those with comorbidities are at higher risk of death
38	Wang Zhang	69	Retrospective Observational	Unknown	548	Not Reported	x						6	Low-dose or no glucocorticoid treatment was associated with a lower hazard compared with high-dose treatment (≥ 1 mg/kg) for 15 days in hospital death
40	Wu Chen	61	Retrospective Observational	Methylprednisolone unknown dose	201	Hospitalized patients	x						4	Treatment with methylprednisolone may be beneficial for patients who develop ARDS
41	Wu Huang	62	Retrospective Observational	Methylprednisolone < 1 mg/kg ED	1763	Severe or critical patients	x						7	Corticosteroid use was not associated with beneficial effect in reducing in-hospital mortality for severe or critical cases in Wuhan
42	Xu Chen	63	Retrospective Observational	Methylprednisolone < 1 mg/kg ED	113	Hospitalized patients	x	x					5	Prolonged SARS-CoV-2 RNA shedding was associated with male sex (P = .009), old age (P = .033), concomitant hypertension (P = .009), delayed admission to hospital after illness onset (P = .001), severe illness at admission (P = .049), invasive mechanical ventilation (P = .006) and corticosteroid treatment (P = .025)
43	Yang Lipes	64	Retrospective Observational	Methylprednisolone, hydrocortisone or dexamethasone > 1 mg/kg ED	15	ICU patients	x				x		6	Possible short-term clinical improvements with corticosteroid. Emphasis the urgent need for high-quality studies on steroids and outcome in critically ill COVID-19 patients
44	Zha Li	65	Retrospective Observational	Methylprednisolone < 1 mg/kg ED	31	Hospitalized patients	x	x	x				5	No evidence of clinical benefit of corticosteroids was found for those without acute respiratory distress syndrome. Virus clearance may be slower in people with chronic HBV infections

^a^M = mortality; V = viral clearance; H = length of hospital stay; R = mechanical ventilator/respirator; O = oxygenation; I = secondary infections

^b^Randomized Embedded Multifactorial Adaptive Platform trial

^c^ED = Prednisolone Equivalent Dose

^d^ Newcastle Ottawa Scale (N.O.S.) for retrospective observational studies. Risk of bias (R.O.B.) for randomized controlled trials: see Fig. 2

Thirty-one of the 44 studies originated in China, 11 in Europe, five in North America, two in South America and one study were multi-continental. The inclusion period of patients ranged from late December 2019 until August 20, 2020. The majority of studies were retrospective observational studies (37/44), five were RCTs [23, 34–37], and there were two studies with historical controls [38, 40]. The study population varied from hospitalized patients (28/44) to patients admitted to the Intensive Care Unit (ICU) (15/44), and one study included discharged patients for viral clearance assessment. The median age of patients ranged from 34 to 75 years.

**Fig. 2:**
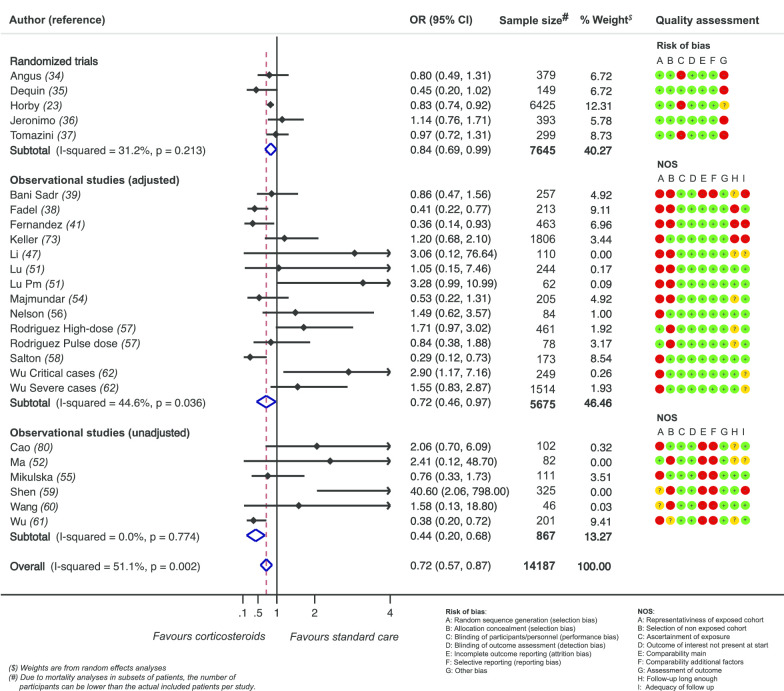
Effect of corticosteroids on mortality

For the observational studies, the median NOS score was 5 (2–8) points (Additional file 5). For the RCT, the risk of bias table is depicted in Fig. 2.

### Corticosteroid regimen (Table 1 and Additional file 6)

In the 44 studies, very diverse corticosteroid strategies were used. If reported (n = 35), methylprednisolone was the most frequently prescribed (n = 28) [35, 36, 38–65]. Prednisone (n = 5) and dexamethasone (n = 5) and hydrocortisone (n = 4) were also used, some in studies that allowed multiple corticosteroid regimens (n = 9).

The indication to start corticosteroids was described in 12 studies (Additional file 6): In three studies, corticosteroids were started at diagnosis/hospital admission. [38, 41, 56] In five studies, ICU admission or respiratory deterioration were the indications to start, either randomized according to study protocol [23, 34, 35, 37] or not randomized [38, 48, 49, 60, 64].

In 29 studies, the dose of corticosteroids was reported: In 16 studies, an equivalent dose of > 1 mg/kg prednisolone was used [37–39, 41, 43, 44, 48–51, 53, 54, 56–58, 64] and in 11 studies a lower equivalent dose than 1 mg/kg prednisolone [23, 34–36, 40, 42, 47, 52, 62, 63, 65]. In two studies, a low- and high-dose group were present [45, 46]. The duration of therapy varied within a range of 5–10 days, in observational studies frequently dependent on clinical condition of patients.

### Effect of steroids on primary and secondary outcomes (Table 2, Additional file 7)

**Table 2:** Summary of findings

Outcomes	Total no events/total no of patients		Relative effect (95% CI)	No of participants (studies)	Certainty of evidence (Grade ^a^)	Comments
	Standard care	Corticosteroids				
Effect of corticosteroids in hospitalized CoVID-19 patients. *Intervention: Corticosteroids; Comparison: Standard of Care*						
In-hospital mortality	1547/9080 (17.0%)	1173/5234 (22.4%)	Estimate 0.72 (0.57–0.87)	14.187^b^ (22)	*RCT:* moderate 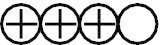 *Non RCT:* Very low 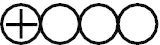	Corticosteroids reduce mortality in CoVID-19 hospitalized patients
Requirement of mechanical ventilation	124/467 (26,6%)	89/472 (18,9%)	Estimate 0.70 (0.54–0.91)	939 (7)	*All studies:* Very low 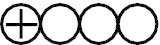	17 studies reported on mechanical ventilation, but effects could only be quantified in 7 studies
Descriptive results: *Data too heterogeneous for quantification of effect*						
Viral Clearance	In corticosteroid group viral clearance time ranged from 10 to 29 days in corticosteroids group and from 8 to 24 days in standard of care group			2.556 (13)	*0* × *RCT* *13* × *retrospective observational study*	Heterogeneous outcome reporting. Corticosteroids are associated with a probable delay in viral clearance
Length of hospital stay	Conflicting results both in favor and against the use of corticosteroids			9.433 (12)	*2* × *RCT,* *10* × *retrospective observational study*	Effect of corticosteroids on length of hospital stay is uncertain
Mechanical ventilation	In 14 out of 17 studies, corticosteroids therapy is associated with beneficial effects on ventilator-free days, on respiratory failure requiring mechanical ventilation and time on mechanical ventilator			12.114 (17)	*5* × *RCT,* *12* × *retrospective observational study*	Beneficial effects of corticosteroids on mechanical ventilation different definitions used)
Oxygenation	Outcome reporting in saturation, p/F ratio and oxygen demand. Conflicting results in favor and against the use of corticosteroids			3.211 (11)	*1* × *RCT,* *10* × *retrospective observational study*	Outcome definition too heterogeneous to draw conclusions
Secondary infections	In five out of six studies, secondary infections and antibiotic use are increased			2.145 (6)	*3* × *RCT* *3* × *retrospective observational study*	Corticosteroids are associated with an increase in infectious complications

^a^ Details on GRADE score are available in Additional file 10

^b^ Due to mortality analyses in subsets of patients, this number of participants is lower than the sum of sample sizes from the included study

Thirty-five of 44 studies reported on *Mortality*. Thirteen of these could not be integrated in the meta-analysis due to only *overall* mortality reporting (n = 5), [45, 63, 64, 66, 67] or only descriptive reporting (n = 8), i.e., of a trend toward better outcome (n = 3), [42, 68, 69], no effect (n = 3) [44, 49, 65] or negative effect on outcome (n = 2) [50, 52]. For the remainder of 22 studies, a pooled estimate was calculated and graphically summarized in a forest plot (Fig. 2). The mortality reported in these studies was mainly 28-day mortality (11 studies), in six studies in-hospital mortality of shorter duration and in five studies there was an unreported follow up period (Additional file 7). The overall risk estimate (OR) was 0.72 (95%CI 0.57–0.87), suggesting a beneficial effect of steroids use in COVID-19 patients hospitalized with moderate or severe respiratory failure on mortality. Studies were heterogeneous (overall I^2^ of 51.1%, p = 0.002) with a between-study variance (tau^2^) of 0.048. For the subset of RCTs, the risk estimate was 0.84 (95%CI 0.72–0.96) and I^2^ and tau^2^ were 31.2% (p = 0.213) and 0.0096, corresponding to less heterogeneity and less between-study variance.

Thirteen of 44 studies reported on *viral clearance*, which most frequently was defined as two consecutive negative RT-PCR on nasopharyngeal swabs, or a cycle time value of 40 or more. In the *corticosteroid* group, viral clearance time ranged from 5 to 29 days, in the *standard of care* group from 8 to 24 days. In nine of 13 studies, viral shedding was delayed in the corticosteroid group. [40, 43, 46, 47, 53, 59, 63, 65, 70] In the other four studies, viral clearance was equal (n = 2) [50, 71] or even better in the corticosteroid group (n = 2) [44, 52]. The numbers are too small to quantify the effect of corticosteroids on viral shedding, or to compare viral shedding duration in subgroups of severity of COVID illness, dose, type or timing of corticosteroids administered. (Additional file 8).

In twelve studies, *length of hospital stay* was compared in corticosteroid versus non-corticosteroid groups. The outcomes varied between studies: six reported longer hospital stay in the corticosteroid group [36, 47, 53, 56, 66] and five reported the opposite [23, 34, 38, 52, 54] or no effect on hospital stay [58].

**Fig. 3:**
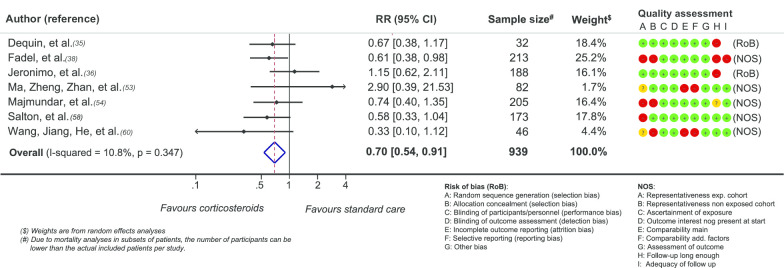
Effect of corticosteroids on need for mechanical ventilation

Fourteen of 17 studies reported a positive effect of corticosteroids on *ventilator-free days* [34, 37, 56], on the *number of patient requiring mechanical ventilation* for respiratory insufficiency [23, 35, 38, 48, 54, 57, 58, 60, 68, 72] or on the *time on mechanical ventilator* [52]. In the pooled analyses fewer patients required mechanical ventilation in the corticosteroids group (RR 0.71 (95%CI 0.54–0.97) (Fig. 3) though only seven studies supplied sufficient data for this analysis. Jeronimo and Keller failed to demonstrate significant differences [36, 73] and one study reported the opposite effect [53]. The dose of corticosteroids could not be related to respiratory outcomes.

Eleven studies reported on the effect of corticosteroids on *oxygenation*. Various definitions were used: liters per minute of oxygen needed, oxygen saturation, PaO_2_/FiO_2_ ratio. The effect of corticosteroids on oxygenation was very heterogeneous: In four studies, there was no significant effect [41, 42, 51, 55], in three studies significant improvement was described [50, 60, 64] and in four studies worse outcome was observed. [35, 39, 54, 57]

Six studies addressed *secondary infections*. More frequently broad spectrum antibiotics were used in the corticosteroid group [39, 47, 53] and more secondary infections or sepsis episodes were described [35, 36]. Only Tomazini found a lower percentage of secondary infections in the corticosteroid group [37]. A dose effect of steroids on development of infections or antibiotic need could not be demonstrated.

## Discussion

In this systematic review and meta-analysis on effectiveness and safety of corticosteroids in COVID-19 patients, the pooled estimate of the observational retrospective studies and the RCTs supported the positive effect of corticosteroids therapy on mortality in COVID-19 disease as first reported in the RECOVERY trial. [23] Furthermore, in already respiratory compromised COVID-19 patients, the need for mechanical ventilation was lower in corticosteroid treated COVID-19 patients. And although data in the studies were too sparse to draw any firm conclusions, there might be a signal of delayed viral clearance and an increase in antibiotic use and infections in the corticosteroid group. However, this did not seem to lead to prolonged hospital stay or increased mortality.

Besides reviews extrapolating knowledge on SARS-CoV or MERS-CoV [21] or on non-viral ARDS [4], or combining studies on SARS-CoV and MERS-CoV [2, 18, 74], to our knowledge, only three other meta-analyses on this subject were conducted with the conflicting results. [24, 75, 76] Sarkar et al. found low‐quality evidence with high variability, showing that in patients with COVID‐19 corticosteroids may be associated with an around twofold increase in mortality [75]. Tlayjeh et al. [76] found no significant difference in mortality or mechanical ventilation need, at the cost of a prolonged viral clearance time. The investigators explained that the discordance in studies was due to bias in the large number of non-RCTs. In the third, very robust, prospective meta-analysis of published and pending trials (inclusion has pretty much stopped since the recovery trial was published), Sterne et al. [24], found that in critically ill patients with COVID-19, the administration of systemic corticosteroids, compared with usual care or placebo, was associated with lower 28-day all-cause mortality. A downside of this rather robust study was that almost 60 percent of the population consisted of the RECOVERY study population and a reasonable amount of data was generated from unpublished, unfinished studies.

Compared to these other systematic reviews on corticosteroids and COVID-19, ours was able to include the largest number of studies and COVID-19 patients. Furthermore, we included both observational studies and RCTs to be able to assess adverse effects such as viral clearance and risk of infections. To obtain the highest possible quality, we excluded non-peer reviewed pre-published manuscripts and furthermore, if available, we included adjusted estimates in the meta-analysis, reducing bias by incongruent study groups (Additional file 9).

Our review has several limitations. The first is that we retrospectively registered our systematic review and meta-analysis. Indeed, it is very important, especially in COVID-19 pandemic times with a high number of publications on COVID-19, to register beforehand to avoid redundancy and inefficiency and to prevent flooding. The review arose from a clinical point of view to gather all literature on corticosteroids and COVID-19, as we clinicians were in doubt whom to administer this drug to. Doing so, we thought it would be best to summarize our findings in a review, since we presumed other clinicians would be struggling with the same questions.

Furthermore, most of the included studies were retrospective cohort studies with increased risk of bias and lower level of evidence, as we confirmed by the GRADE classification (Table 2, Additional file 10). Besides that, large heterogeneity in the studies was present (i.e., study population, type, dose, initiation and duration of corticosteroids and outcome measures) and we emphasize that definitions of primary and secondary outcome measures varied substantially per included article and pooled data from this review should be interpreted cautiously. However, we decided beforehand to only include short-term mortality, i.e., 28-day or closely related short-term in-hospital mortality. Furthermore, we decided to carefully note the applied definitions in the studies in our data extraction tables and include only outcomes as defined by the investigators if they filled our inclusions criteria. We agree that the remaining variation in definitions is indeed a drawback of this review. And although the pooled data from this review should therefore be interpreted cautiously, they represent the effect of corticosteroids on short-term 28-day mortality and the pooled estimates for RCTs and adjusted and unadjusted observational studies pointed toward the same direction, i.e., of a beneficial effect. In many studies, confounding by indication was evidently present: two studies described that corticosteroid administration was “at the discretion of the treating physician” [40, 41] and four reported that severe patients were more likely to receive corticosteroid treatment. [40, 49, 60, 66] Many studies had incomplete follow-up and a considerable amount of patients did not reach definite endpoints. However, our conscious exclusion of non-peer-reviewed studies, the focus on a measurable and quantifiable endpoint, and, if possible, inclusion of risk estimates corrected for confounders and propensity matched, increased the validity of the retrospective evidence supporting the RECOVERY trial. Furthermore, from the included studies, 26 originated in China, with 13 from the hotspot regions (Wuhan, Hubei and Shanghai). This might impair generalizability but although overlapping study populations were present within the included studies (see table in Additional file 4.), this was only incidentally the case for secondary outcome measures. For the main outcome, multiple publication bias was unlikely (Additional file 11). Furthermore, 42% of the study population was included from outside China. Moreover, in terms of generalizability, the median age from the included patients in this review ranged from 34 to 72 years. However, data from the CDC state that 42.9% of hospitalized patients in the USA are > 65 years and European numbers from the European Centre for Disease Prevention and Control (ECDC) show that 54.2% hospitalized patients are > 65 years with great variation between countries. [77, 78] Despite aforementioned limitations, still, this systematic review and meta-analysis confirms the conclusion of the meta-analysis of the RCTs that critically ill COVID-19 patients hospitalized for moderate or severe respiratory failure, with or without mechanical ventilation, should receive corticosteroids.

Severe COVID-19 patients are faced with a twofold problem. On the one hand, there is the hyperinflammatory response, resulting in pulmonary thrombosis, extravasation of cell debris and acute lung injury or even ARDS [79]. On the other hand, there is a need to clear the viral infection itself. This primary phenomenon suggests a possible target for corticosteroids [17]. Thus, the confirmation that there is predominantly a beneficial effect of corticosteroids on mortality is congruent with pathophysiological reasoning and prior knowledge. In our study we, found a signal of delayed viral clearance, but data in the studies were too sparse to draw any firm conclusions. Therefore, what is lacking is knowledge on the optimal start of corticosteroid administration after the start of illness, specific subpopulations and type, dose and duration of corticosteroids. RCTs so far reported a strongly beneficial effect on mortality but did not investigate optimal timing and indication of corticosteroid administration [24], and our study was not able to provide an answer to the latter issues, either. Therefore, future research should focus on which patient characteristics, laboratory and radiological markers can be used to guide indication and timing of corticosteroid treatment, particularly in relation to safety (e.g., delayed viral clearance and increased incidence of secondary infections).

## Conclusion

Our findings from both observational studies and RCTs confirm a beneficial effect of corticosteroids on short-term mortality and a reduction in the need for mechanical ventilation. And although data in the studies were too sparse to draw any firm conclusions, there might be a signal of delayed viral clearance and an increase in secondary infections related to corticosteroid use. Optimal timing, dose and duration of corticosteroids, in relation to safety, remain subject for further investigation. Since corticosteroids are affordable and easily accessible in healthcare systems quivering under the pressure of the global outbreak of this rapidly spreading coronavirus, this field of research should be a universal priority.

## Supplementary information

**Additional file 1**. Search strategy.

**Additional file 2**. Data extraction form.

**Additional file 3**. Excluded references.

**Additional file 4**. Data extraction General Information.

**Additional file 5**. NOS score.

**Additional file 6**. Data extraction Treatment.

**Additional file 7 **. Data extraction Outcome.

**Additional file 8**. Viral Clearance time.

**Additional file 9**. Mechanical ventilation.

**Additional file 10**. Grade classification.

**Additional file 11**. Population bias.

## Data Availability

Not applicable.

## Abbreviations

ARDS: Acute respiratory distress syndrome
CDC: Centers for Disease Control and Prevention
CI: Confidence interval
COVID-19: Coronavirus disease 2019
CT: Computed tomography
ECDC: European Centre for Disease Prevention and Control
FiO2: Inspiratory oxygen fraction
HR: Hazard ratio
ICU: Intensive care Unit
IQR: Interquartile range
LOS: Length of stay
MERS-CoV: Middle East respiratory syndrome coronavirus
OR: Odds ratio
NOS: Newcastle Ottawa Scale
NR: Not reported
OR: Odds ratio
PaO2: Arterial oxygen tension
PRISMA: Preferred Reporting Items for Systematic Reviews and Meta-Analyses
RNA: Ribonucleic acid
RR: Rate ratio
RT-PCR: Reverse transcription polymerase chain reaction
SARS-CoV: Severe acute respiratory syndrome coronavirus
SD: Standard deviation
Steroids: Glucocorticoids or corticoids
SpO2: Plasma oxygen saturation
WHO: World Health Organization
