# Supplementary material for: Corticosteroid use in COVID-19 patients: a systematic review and meta-analysis on clinical outcomes
Source: Crit Care. 2020 Dec 14;24:696. doi: 10.1186/s13054-020-03400-9 (PMC7735177; doi:10.1186/s13054-020-03400-9)
Supplement: Supplementary file 1 — Additional file 1. Search strategy. [file 13054_2020_3400_MOESM1_ESM.docx]

**Supplement 1. Search strategy**

**PubMed:**

((("adrenal cortex hormones"[Mesh] OR adrenal cortex hormone*[tw] OR "glucocorticoids"[Pharmacological Action] OR corticoid[tw] OR corticoids[tw] OR glucocorticoid*[tw] OR steroids[tw] OR steroid[tw] OR corticosteroid*[tw] OR "cortico steroid"[tw] OR "cortico steroids"[tw] OR prednison*[tw] OR dexamethason*[tw] OR beclomethason*[tw] OR cortison[tw] OR cortisone[tw] OR cortisons[tw] OR cortisones[tw] OR prednisolon*[tw] OR budenosid*[tw] OR alclometason*[tw] OR betamethason*[tw] OR Celeston*[tw] OR ciclesonid*[tw] OR Clobetasol*[tw] OR clobetason*[tw] OR clocortolon*[tw] OR desoximethason*[tw] OR Desoximetason*[tw] OR dichlorison*[tw] OR diflorason*[tw] OR Diflucortolon*[tw] OR difluprednat*[tw] OR drocinonid*[tw] OR Flumethason*[tw] OR flumetason*[tw] OR "Fluocinolone Acetonide"[tw] OR Fluocinonid*[tw] OR Fluocort*[tw] OR Fluorometholon*[tw] OR fluperolon*[tw] OR flupredni*[tw] OR Flurandrenol*[tw] OR Fluticason*[tw] OR FX006[tiab] OR halometason*[tw] OR medryson*[tw] OR Melengestrol*[tw] OR Methylpredni*[tw] OR Paramethason*[tw] OR prednicarbate*[tw] OR rimexolon*[tw] OR terofenamat*[tw] OR Tobramycin*[tw] OR Triamcinolon*[tw] OR deltason*[tw] OR hydrocortisone*[tw] OR dehydrocortison*[tw] OR 17-Ketosteroid*[tw] OR Androstenedion*[tw] OR Androsteron*[tw] OR Dehydroepiandrosteron*[tw] OR Estrone[tw] OR Etiocholanolon*[tw] OR Hydroxycorticosteroid*[tw] OR Desoxycorticosteron*[tw] OR pregnenolon*[tw] OR hydropregnenolon*[tw] OR rectodelt[tw] OR sterapred[tw] OR ultracorten[tw] OR cortan[tiab] OR cortancyl[tw] OR panafcort[tw] OR cutason[tw] OR decortin[tw] OR dacortin[tw] OR decortisyl[tw] OR encorton*[tw] OR enkortolon[tw] OR kortancyl[tw] OR meticorten[tw] OR Orasone[tw] OR panasol[tw] OR predni[tw] OR VB0R961HZT[tw] OR pronison*[tw] OR "Depo-Medrol"[tw] OR hexadecadrol*[tw] OR dexamethasone*[tw] OR dexason*[tw] OR millicorten[tw] OR dexpak[tw] OR maxidex[tw] OR oradexon*[tw] OR decaject[tw] OR hexadrol[tw] OR 7S5I7G3JQL[tw] OR beclometason*[tw] OR ascocortonyl[tw] OR beclocort[tw] OR beconase[tw] OR beclomet[tw] OR bronchocort[tw] OR mometason*[tw] OR fludrocortisone*[tw] OR budesonide*[tw] OR cortisol[tw] OR cortisoles[tw] OR cortisols[tw] OR sofradex[tw] OR entocort[tw]) AND ("COVID-19"[Supplementary Concept] OR "severe acute respiratory syndrome coronavirus 2"[Supplementary Concept] OR "severe acute respiratory syndrome cov 2"[all fields] OR "severe acute respiratory syndrome cov2"[all fields] OR COVID-19*[all fields] OR COVID19*[all fields] OR COVID2019*[all fields] OR COVID 2019*[all fields] OR "acute respiratory syndrome coronavirus 2"[all fields] OR SARS-COV*[all fields] OR SARSCOV*[all fields] OR 2019ncov*[all fields] OR 2019 ncov*[all fields] OR ncov 2019*[all fields] OR ncov2019*[all fields] OR novel coronavir*[all fields] OR novel corona*[all fields] OR new corona*[tw] OR sars2[all fields] OR "sars coronavirus 2"[all fields] OR "sars corona virus 2"[all fields] OR ((coronavir*[all fields] OR corona[all fields] OR covid[all fields] OR cov[all fields] OR ncov[all fields])) NOT (("COVID-19"[Supplementary Concept] OR "severe acute respiratory syndrome coronavirus 2"[Supplementary Concept] OR "severe acute respiratory syndrome cov 2"[all fields] OR "severe acute respiratory syndrome cov2"[all fields] OR COVID-19*[all fields] OR COVID19*[all fields] OR COVID2019*[all fields] OR COVID 2019*[all fields] OR "acute respiratory syndrome coronavirus 2"[all fields] OR SARS-COV*[all fields] OR SARSCOV*[all fields] OR 2019ncov*[all fields] OR 2019 ncov*[all fields] OR ncov 2019*[all fields] OR ncov2019*[all fields] OR novel coronavir*[all fields] OR novel corona*[all fields] OR new corona*[tw] OR sars2[all fields] OR "sars coronavirus 2"[all fields] OR "sars corona virus 2"[all fields] OR ((coronavir*[all fields] OR corona[all fields] OR covid[all fields] OR cov[all fields] OR ncov[all fields]))) AND ("Intensive Care Units"[Mesh] OR ICU[tiab] OR ICUs[tiab] OR "ICU's"[tiab] OR intensive care*[tw] OR "Critical Care"[Mesh] OR critical care*[tw] OR "Emergency Treatment"[Mesh] OR emergency care*[tw] OR emergency treatment*[tw] OR "Intubation"[Mesh] OR intubation*[tw] OR intubating[tw] OR intubate*[tw] OR "Critical Illness"[Mesh] OR critical ill*[tw] OR critically ill*[tw]))) AND ("2019/12/01"[PDat] : "3000/12/31"[PDat])

**Embase:**

(exp corticosteroid/ OR exp glucocorticoid/ OR corticoid* OR glucocorticoid* OR steroid OR steroids OR corticosteroid* OR "cortico steroid*" OR prednison* OR dexamethason* OR beclomethason* OR cortison OR cortisone OR cortisons OR cortisones OR prednisolon* OR budenosid* OR alclometason* OR betamethason* OR Celeston* OR ciclesonid* OR Clobetasol* OR clobetason* OR clocortolon* OR desoximethason* OR Desoximetason* OR dichlorison* OR diflorason* OR Diflucortolon* OR difluprednat* OR drocinonid* OR Flumethason* OR flumetason* OR "Fluocinolone Acetonide" OR Fluocinonid* OR Fluocort* OR Fluorometholon* OR fluperolon* OR flupredni* OR Flurandrenol* OR Fluticason* OR FX006 OR halometason* OR medryson* OR Melengestrol* OR Methylpredni* OR Paramethason* OR prednicarbate* OR rimexolon* OR terofenamat* OR Tobramycin* OR Triamcinolon* OR deltason* OR hydrocortisone* OR dehydrocortison* OR 17-Ketosteroid* OR Androstenedion* OR Androsteron* OR Dehydroepiandrosteron* OR Estrone OR Etiocholanolon* OR Hydroxycorticosteroid* OR Desoxycorticosteron* OR pregnenolon* OR hydropregnenolon* OR rectodelt OR sterapred OR ultracorten OR cortan OR cortancyl OR panafcort OR cutason OR decortin OR dacortin OR decortisyl OR encorton* OR enkortolon OR kortancyl OR meticorten OR Orasone OR panasol OR predni OR VB0R961HZT OR pronison* OR "Depo-Medrol" OR hexadecadrol* OR dexamethasone* OR dexason* OR millicorten OR dexpak OR maxidex OR oradexon* OR decaject OR hexadrol OR 7S5I7G3JQL OR beclometason* OR ascocortonyl OR beclocort OR beconase OR beclomet OR bronchocort OR mometason* OR fludrocortisone* OR budesonide* OR cortisol OR cortisoles OR cortisols OR sofradex OR entocort) AND ("severe acute respiratory syndrome*" OR exp Coronavirinae/ OR exp coronavirus infections/ OR coronaviri* OR betacoronavir* OR MERS OR "middle East respiratory" OR "COVID-19*" OR exp sars-related coronavirus/ OR COVID19* OR COVID2019* OR "acute respiratory syndrome coronavirus 2" OR SARS OR SARSCOV* OR 2019ncov* OR "ncov2019*" OR sars2 OR coronavir* OR corona OR covid OR cov OR ncov) AND (exp Intensive Care/ OR ICU OR ICUs OR "ICU's" OR "intensive care*" OR "critical care*" OR exp Emergency Treatment/ OR "emergency care*" OR "emergency treatment*" OR exp Intubation/ OR intubation* OR intubating OR intubate* OR exp Critical Illness/ OR "critical ill*" OR "critically ill*")

**Web of Science:**

(corticoid* OR glucocorticoid* OR steroid OR steroids OR corticosteroid* OR "cortico steroid*" OR prednison* OR dexamethason* OR beclomethason* OR cortison OR cortisone OR cortisons OR cortisones OR prednisolon* OR budenosid* OR alclometason* OR betamethason* OR Celeston* OR ciclesonid* OR Clobetasol* OR clobetason* OR clocortolon* OR desoximethason* OR Desoximetason* OR dichlorison* OR diflorason* OR Diflucortolon* OR difluprednat* OR drocinonid* OR Flumethason* OR flumetason* OR "Fluocinolone Acetonide" OR Fluocinonid* OR Fluocort* OR Fluorometholon* OR fluperolon* OR flupredni* OR Flurandrenol* OR Fluticason* OR FX006 OR halometason* OR medryson* OR Melengestrol* OR Methylpredni* OR Paramethason* OR prednicarbate* OR rimexolon* OR terofenamat* OR Tobramycin* OR Triamcinolon* OR deltason* OR hydrocortisone* OR dehydrocortison* OR 17-Ketosteroid* OR Androstenedion* OR Androsteron* OR Dehydroepiandrosteron* OR Estrone OR Etiocholanolon* OR Hydroxycorticosteroid* OR Desoxycorticosteron* OR pregnenolon* OR hydropregnenolon* OR rectodelt OR sterapred OR ultracorten OR cortan OR cortancyl OR panafcort OR cutason OR decortin OR dacortin OR decortisyl OR encorton* OR enkortolon OR kortancyl OR meticorten OR Orasone OR panasol OR predni OR VB0R961HZT OR pronison* OR "Depo-Medrol" OR hexadecadrol* OR dexamethasone* OR dexason* OR millicorten OR dexpak OR maxidex OR oradexon* OR decaject OR hexadrol OR 7S5I7G3JQL OR beclometason* OR ascocortonyl OR beclocort OR beconase OR beclomet OR bronchocort OR mometason* OR fludrocortisone* OR budesonide* OR cortisol OR cortisoles OR cortisols OR sofradex OR entocort) AND ("severe acute respiratory syndrome*" OR coronaviri* OR betacoronavir* OR MERS OR "middle East respiratory" OR "COVID-19*" OR COVID19* OR COVID2019* OR "acute respiratory syndrome coronavirus 2" OR SARS OR SARSCOV* OR 2019ncov* OR "ncov2019*" OR sars2 OR coronavir* OR corona OR covid OR cov OR ncov) AND (ICU OR ICUs OR "ICU's" OR "intensive care*" OR "critical care*" OR "emergency care*" OR "emergency treatment*" OR intubation* OR intubating OR intubate* OR "critical ill*" OR "critically ill*")
