# Supplementary material for: Corticosteroid use in COVID-19 patients: a systematic review and meta-analysis on clinical outcomes
Source: Crit Care. 2020 Dec 14;24:696. doi: 10.1186/s13054-020-03400-9 (PMC7735177; doi:10.1186/s13054-020-03400-9)
Supplement: Supplementary file 2 — Additional file 2. Data extraction form. [file 13054_2020_3400_MOESM2_ESM.docx]

# **Supplement 2. Data extraction form.**

1. **General**

| **Author** | **Country, region** | **Hospital** | **Publication date** | **Period of inclusion** | **Study type** | **Sample size** | **Follow-up (days)** | **Study population** | **Study groups** | **Mean/median age, in study groups (yr)** | **Males (%), in study groups** | **Critically ill patients: (%)** | **NOS** |
| --- | --- | --- | --- | --- | --- | --- | --- | --- | --- | --- | --- | --- | --- |

1. **Treatment**

| **Author** | **Type of steroid** | **Patients treated (number, %)**  **Describe subsets** | **Indication** | **Dose (mg/d)**  **Prednisolone ED** | **Initiation of treatment (days) relative to start of symptoms/hospital admission** (has to be specified ) | **Duration of treatment (days)** |
| --- | --- | --- | --- | --- | --- | --- |

1. **Outcome**

| **Author** | **Mortality Definition**  (short-term, e.g 28-day, 30-day, in-hospital, overall) | **Mortality (%)**  (quantification) | **Effect on Mortality**  (summary described in words) | **Viral Clearance**  **Definition**  (i.e. according to RT-PCR in respiratory specimens) | **Viral clearance (days)**  **(**quantification) | **Effect on viral clearance**  (summary described in words) | **Effect on Length of hospital stay (days)** | **Effect on need for requirement of mechanical ventilation (%)** | **Oxygenation Definition**  (as defined by the investigators) | **Effect on oxygenation** (quantification) | **Effect on respiratory secondary outcomes**  (duration of mechanical ventilation, ventilator free days) | **Effect on secondary infection** |
| --- | --- | --- | --- | --- | --- | --- | --- | --- | --- | --- | --- | --- |
