# Supplementary material for: Corticosteroid use in COVID-19 patients: a systematic review and meta-analysis on clinical outcomes
Source: Crit Care. 2020 Dec 14;24:696. doi: 10.1186/s13054-020-03400-9 (PMC7735177; doi:10.1186/s13054-020-03400-9)
Supplement: Supplementary file 3 — Additional file 3. Excluded references. [file 13054_2020_3400_MOESM3_ESM.docx]

**Supplement 3. Excluded references after full text screening.**

1. CME Exam 1: Corticosteroids, But Not TNF Antagonists, Are Associated with Adverse COVID-19 Outcomes in Patients With Inflammatory Bowel Diseases: Results from an International Registry. Gastroenterology. 2020;159(2):e13-e4.

2. Akbudak IH. Corticosteroid Therapy in COVID-19 Disease. J Crit Intensive Care. 2020;11:30-1.

3. Ambika R, Seelakshmi M. A review on the use of dexamethasone in covid-19. International Journal of Pharmaceutical Sciences Review and Research. 2020;63:202-5.

4. Arca KN, Smith JH, Chiang CC, Starling AJ, Robertson CE, Halker Singh RB, et al. COVID-19 and Headache Medicine: A Narrative Review of Non-Steroidal Anti-Inflammatory Drug (NSAID) and Corticosteroid Use. Headache. 2020.

5. Blasco Patino F, Guillamon Sanchez AE. Indication of glucocorticoid pulses for patients with SARS-CoV-2 infection. Med Clin (Barc). 2020.

6. Boglione L, Rostagno R, Poletti F, Moglia R, Bianchi B, Esposito M, et al. The proper use of corticosteroids for 2019-nCov pneumonia: Towards promising results? J Infect. 2020:4781.

7. Brotherton H, Usuf E, Nadjm B, Forrest K, Bojang K, Samateh AL, et al. Dexamethasone for COVID-19: data needed from randomised clinical trials in Africa. The Lancet Global Health. 2020;8(9):e1125-e6.

8. Chen J, Bai H, Liu J, Chen G, Liao Q, Yang J, et al. Distinct clinical characteristics and risk factors for mortality in female COVID-19 inpatients: a sex-stratified large-scale cohort study in Wuhan, China. Clin Infect Dis. 2020.

9. Chen L, Zhang B, Ti MN, Yang K, Zou Y, Zhang S. Clinical course of severe and critically ill patients with coronavirus disease 2019 (COVID-19): A comparative study. J Infect. 2020;81(2):e82-e4.

10. Chrousos GP, Meduri GU. Critical COVID-19 disease, homeostasis, and the "surprise" of effective glucocorticoid therapy. Clin Immunol. 2020;219:108550.

11. Cohen SP, Baber ZB, Buvanendran A, McLean BC, Chen Y, Hooten WM, et al. Pain Management Best Practices from Multispecialty Organizations During the COVID-19 Pandemic and Public Health Crises. Pain Med. 2020;21(7):1331-46.

12. Conticini E, Franchi F, Bennett D, Valente S, Mazzei MA, Bargagli E, et al. High dosage of methylprednisolone as a rescue, second-line treatment in COVID-19 patients who failed to respond to tocilizumab. Ann Rheum Dis. 2020.

13. Corral L, Bahamonde A, Arnaiz delas Revillas F, Gomez-Barquero J, Abadia-Otero J, Garcia-Ibarbia C, et al. GLUCOCOVID: A controlled trial of methylprednisolone in adults hospitalized with COVID-19 pneumonia. medRxiv. 2020:2020.06.17.20133579.

14. D'Ardes D, Pontolillo M, Esposito L, Masciarelli M, Boccatonda A, Rossi I, et al. Duration of COVID-19: Data from an Italian Cohort and Potential Role for Steroids. Microorganisms. 2020;8(9).

15. Delafosse M, Saint-Jaques C, Petit-Hoang C, François H, Peltier J, Nicolet L, et al. Steroids: A Therapeutic Option for COVID-19 Pneumonia Patients With ESRD? Kidney International Reports. 2020;5(8).

16. Dubernet A, Larsen K, Masse L, Allyn J, Foch E, Bruneau L, et al. A comprehensive strategy for the early treatment of COVID-19 with azithromycin/hydroxychloroquine and/or corticosteroids: Results of a retrospective observational study in the French overseas department of Reunion Island. J Glob Antimicrob Resist. 2020;23:1-3.

17. Goursaud S, Descamps R, Daubin C, du Cheyron D, Valette X. Corticosteroid use in selected patients with severe acute respiratory distress syndrome related to COVID-19. J Infect. 2020;81(2):e89-e90.

18. Hu F, Yin G, Chen Y, Song J, Ye M, Liu J, et al. Corticosteroid, oseltamivir and delayed admission are independent risk factors for prolonged viral shedding in patients with Coronavirus Disease 2019. Clin Respir J. 2020.

19. Huang Q, Deng X, Li Y, Sun X, Chen Q, Xie M, et al. Clinical characteristics and drug therapies in patients with the common-type coronavirus disease 2019 in Hunan, China. Int J Clin Pharm. 2020;42(3):837-45.

20. Huang Q, Wu X, Zheng X, Luo S, Xu S, Weng J. Targeting inflammation and cytokine storm in COVID-19. Pharmacol Res. 2020;159:105051.

21. Jin S, Ronghui D, Qiaofa L, Jianhong W, Shabei X, Zhenghua K, et al. The corticosteroid treatment and response of patients with COVID-19 in Hubei, China: a retrospective, cohort study. Research Square. 2020.

22. John TM, Malek AE, Mulanovich VE, Adachi JA, Raad II, Hamilton AR. Migratory Pulmonary Infiltrates in a Patient With COVID-19 Infection and the Role of Corticosteroids. Mayo Clin Proc. 2020;95(9):2038-9.

23. Kevorkian JP, Riveline JP, Vandiedonck C, Girard D, Galland J, Feron F, et al. Early short-course corticosteroids and furosemide combination to treat non-critically ill COVID-19 patients: An observational cohort study. J Infect. 2020:4825.

24. Khamis F, Al-Zakwani I, Al Naamani H, Al Lawati S, Pandak N, Omar MB, et al. Clinical characteristics and outcomes of the first 63 adult patients hospitalized with COVID-19: An experience from Oman. J Infect Public Health. 2020;13(7):906-13.

25. Lammers T, Sofias AM, van der Meel R, Schiffelers RM, Storm G, Tackle F, et al. Decamethasone nanomedicines for COVID-19. Nature Nanotechnology 2020;15(8):622-4.

26. Lennox JL. Methylprednisolone for COVID-19: Was Benjamin Rush prescient? Clin Infect Dis. 2020.

27. Li S, Hu Z, Song X. High-dose but Not Low-dose Corticosteroids Potentially Delay Viral Shedding of Patients With COVID-19. Clinical Infectious Diseases. 2020.

28. Li T-Z, Cao Z-H, Chen Y, Cai M-T, Zhang L-Y, Xu H, et al. Duration of SARS-CoV-2 RNA shedding and factors associated with prolonged viral shedding in patients with COVID-19. Journal of medical virology. 2020:10.1002/jmv.26280.

29. Lian J, Jin X, Hao S, Jia H, Cai H, Zhang X, et al. Epidemiological, clinical, and virological characteristics of 465 hospitalized cases of coronavirus disease 2019 (COVID-19) from Zhejiang province in China. Influenza Other Respir Viruses. 2020;14(5):564-74.

30. Lim MA, Pranata R. Worrying situation regarding the use of dexamethasone for COVID-19. Ther Adv Respir Dis. 2020;14:1753466620942131.

31. Ling Y, Xu S-B, Lin Y-X, Tian D, Zhu Z-Q, Dai F-H, et al. Persistence and clearance of viral RNA in 2019 novel coronavirus disease rehabilitation patients. Chin Med J (Engl). 2020;133(9):1039-43.

32. Lipworth B, Kuo CR, Lipworth S, Chan R. Inhaled Corticosteroids and COVID-19. Am J Respir Crit Care Med. 2020;202(6):899-900.

33. Liu F, Ji C, Luo J, Wu W, Zhang J, Zhong Z, et al. Clinical characteristics and corticosteroids application of different clinical types in patients with corona virus disease 2019. Sci Rep. 2020;10(1):13689.

34. Mo P, Xing Y, Xiao Y, Deng L, Zhao Q, Wang H, et al. Clinical characteristics of refractory COVID-19 pneumonia in Wuhan, China. Clinical Infectious Diseases. 2020.

35. Moon C. Dexamethasone to the rescue. Nature Reviews Immunology. 2020;20(8):463-.

36. Murohashi K, Hagiwara E, Kitayama T, Yamaya T, Higa K, Sato Y, et al. Outcome of early-stage combination treatment with favipiravir and methylprednisolone for severe COVID-19 pneumonia: A report of 11 cases. Respir Investig. 2020.

37. Nicolau DV, Bafadhel M. Inhaled corticosteroids in virus pandemics: a treatment for COVID-19? The Lancet Respiratory Medicine. 2020;8(9):846-7.

38. Patel SK, Saikumar G, Rana J, Dhama J, Yatoo MI, Tiwari R, et al. Dexamethasone: A boon for critically ill COVID-19 patients? Travel Med Infect Dis. 2020;37:101844.

39. Prescott HC, Rice TW. Corticosteroids in COVID-19 ARDS: Evidence and Hope During the Pandemic. JAMA. 2020;324(13):1292-5.

40. Sarkar S, Khanna P, Soni KD. Are the steroids a blanket solution for COVID-19? A systematic review and meta-analysis. J Med Virol. 2020.

41. Sharun K, Tiwari R, Dhama J, Dhama K. Diagnosis and treatment of coronavirus disease 2019 (COVID-19): Laboratory, PCR, and chest CT imaging findings. Int J Surg. 2020;79:143-53.

42. Siemieniuk RA, Bartoszko JJ, Ge L, Zeraatkar D, Izcovich A, Kum E, et al. Drug treatments for covid-19: living systematic review and network meta-analysis. BMJ. 2020;370:m2980.

43. Sterne JAC, Murthy S, Diaz JV, Slutsky AS, Villar J, Angus DC, et al. Association Between Administration of Systemic Corticosteroids and Mortality Among Critically Ill Patients With COVID-19: A Meta-analysis. JAMA. 2020.

44. Varma A, Kosuri S, Ustun C, Ibrahim U, Moreira J, Bishop MR, et al. COVID-19 infection in hematopoietic cell transplantation: age, time from transplant and steroids matter. Leukemia. 2020;34(10):2809-12.

45. Wang D, Hu B, Hu C, Zhu F, Liu X, Zhang J, et al. Clinical Characteristics of 138 Hospitalized Patients With 2019 Novel Coronavirus–Infected Pneumonia in Wuhan, China. JAMA. 2020;323(11):1061-9.

46. Wang J, Yang Q, Zhang P, Sheng J, Zhou J, Qu T. Clinical characteristics of invasive pulmonary aspergillosis in patients with COVID-19 in Zhejiang, China: a retrospective case series. Crit Care. 2020;24(1):299.

47. Wang K, Tan F, Zhou R, Liu D, Ni Z, Liu J, et al. Therapeutic response to corticosteroids in a critically ill patient with COVID-19: A case report. Medicine (Baltimore). 2020;99(31):e21597.

48. Wang Y, Lu X, Li Y, Chen H, Chen T, Su N, et al. Clinical Course and Outcomes of 344 Intensive Care Patients with COVID-19. Am J Respir Crit Care Med. 2020;201(11):1430-4.

49. Winthrop K, Mariette X. To immunosippress: whom, when and how? That is the question with COVID-19. Ann Rheum Dis. 2020;79 1129-31.

50. Yamamura H, Matsuura H, Nakagawa J, Fukuoka H, Domi H, Chujoh S. Effect of favipiravir and an anti-inflammatory strategy for COVID-19. Crit Care. 2020;24(1):413.

51. Yamasaki Y, Ooka S, Tsuchida T, Nakamura Y, Hagiwara Y, Naitou Y, et al. The peripheral lymphocyte count as a predictor of severe COVID-19 and the effect of treatment with ciclesonide. Virus Res. 2020:198089.

52. Yang Q, Xie L, Zhang W, Zhao L, Wu H, Jiang J, et al. Analysis of the clinical characteristics, drug treatments and prognoses of 136 patients with coronavirus disease 2019. J Clin Pharm Ther. 2020;45(4):609-16.

53. Zachariah U, Nair SC, Goel A. Targeting Raised von Willebrand Factor Levels in Liver Diseases: Opening Up Newer Therapeutic Avenues. EMJ Hepatology. 2020.

54. Zhang G, Hu C, Luo L, Fang F, Chen Y, Li J, et al. Clinical features and short-term outcomes of 221 patients with COVID-19 in Wuhan, China. Journal of Clinical Virology. 2020;127:104364.

55. Zhao J, Gao HY, Feng ZY, Wu QJ. A Retrospective Analysis of the Clinical and Epidemiological Characteristics of COVID-19 Patients in Henan Provincial People's Hospital, Zhengzhou, China. Front Med (Lausanne). 2020;7:286.

56. Zhou F, Yu T, Du R, Fan G, Liu Y, Liu Z, et al. Clinical course and risk factors for mortality of adult inpatients with COVID-19 in Wuhan, China: a retrospective cohort study. The Lancet. 2020;395.

57. Zhou W, Liu Y, Tian D, Wang C, Wang S, Cheng J, et al. Potential benefits of precise corticosteroids therapy for severe 2019-nCoV pneumonia. Signal Transduct Target Ther. 2020;5(1):18.
