# Supplementary material for: Corticosteroid use in COVID-19 patients: a systematic review and meta-analysis on clinical outcomes
Source: Crit Care. 2020 Dec 14;24:696. doi: 10.1186/s13054-020-03400-9 (PMC7735177; doi:10.1186/s13054-020-03400-9)
Supplement: Supplementary file 4 — Additional file 4. Data extraction General Information. [file 13054_2020_3400_MOESM4_ESM.docx]

**Supplement 4. Data extraction of General information.**

| **Author (ref)** | **Country, region** | **Hospital** | **Publication date** | **Period of inclusion** | **Study type** | **Sample size** | **Follow-up (days)** | **Study population** | **Study groups** | **Mean/median age, in study groups (yr)** | **Males (%), in study groups** | **Critically ill patients: (%)** | **NOS** |
| --- | --- | --- | --- | --- | --- | --- | --- | --- | --- | --- | --- | --- | --- |
| **Angus REMAP-CAP**  **34** | Multiple countries in Europe, USA, Australia, New Zealand, Canada, | multiple | September 2, 2020 | March 2020 – June 17, 2020 | Randomized, Embedded, Multifactoria, adaptive Platform Trial | 403 | 21 days | Adults > 18 years admitted to ICU for provision of respiratory or cardiovascular organ support. | Fixed 7 day course (50-100 mg Hydrocortisone every 6 hours)  versus  Shock-dependent dose (50 mg Hydrocortisone every 6 hours when shock was clinically evident)  versus  no steroids. | Fixed 7 days:  Mean 60,4  Shock  Mean 59,5  No steroids  59,9 | Fixed 7 days: 61%  Shock: 57%  No steroids: 71 % | all | RCT, NA |
| **Bani Sadr**  **39** | Reims France | University hospital of Reims | July 4, 2020 | March 3 – April 14 2020 | Cohort before/after-study  Corticosteroids were  were widely used after 27 March  Study design compares period ‘before’ March 27 and ‘after’ | 319 | until death, until ICU admission or death.  Median 16 days. | Adult patients admitted at the hospital for CoVID | Before: regular care  After: prednisone 1 mg/kg or Methylprednisolone equivalent | Before: 70.1  After: 71,8 | Before 54%  After 51,7% | Before 34 %  After 23,6% | 8 |
| **Cao et al.**  **80** | China, Wuhan | Zhongnan Hospital | 2 April 2020 | 3 January to 1 February, 2020 | Retrospective observational | 102 | Followed up until discharge | Hospitalized COVID-19 patients | Non-survivors (17) versus survivors (85) | 54 | 52% | Transfer to ICU: 17.6% | 6 |
| **Chen Zu Zu**  **70** | China, Guangzhou | Guanzhou Eighth people’s Hospital | June 26, 2020 | January 20 – March 15 2020 | Retrospective | 267 | Until discharge (longest before discharge was 45 days) | Symptomatic COVID-19 patients | No group division | all patients 49 (34-62) | all patients 45.3% | 1.5% | 7 |
| **Chroboczek et al.**  **72** | France | Centre Hospitalier Alpes Léman | 16 June 2020 | 10 March to 9 April, 2020 | Retrospective observational | 70 | NR | Hospitalized COVID-19 patients requiring more than 3L of oxygen | Steroid 21 non-steroid 49 | NR | NR | NR | 6 |
| ***Dequin***  ***(CAPE COVID)***  ***35*** | *France* | Multicentre | September 2, 2020 | March 7, 2020- June1, 2020 | Randomized controlled trial | 149 | 21 days | Adult patients admitted to the ICU with Respiratory failure  One of 4 severity  criteria had to be present: need formechanical ventilation with  a positive end-expiratory pressure (PEEP) of 5 cm H20 ormore;  a ratio of PaO2 to fraction of inspired oxygen (FIo2) less than  300 on high-flow oxygen therapy with an FIO2 value of at least  50%; for patients receiving oxygen through a reservoir mask,  a PaO2:FIO2 ratio less than 300, estimated using prespecified  charts; or a Pulmonary Severity Index18 greater than 130. | Hydrocortisone versus  placebo. | Steroids 63.1  Placebo 66.3 | Steroids: 71%  Placebo: 68,5 % | all | RCT, na |
| ***Fadel et al.***  ***38*** | *USA, Michigan* | Five hospitals in southeast and south-central Michigan | 19 May 2020 | 12 March to 27 March 2020 | Quasi-experimental | 213 | ≥14 days | Adult hospitalized patients with confirmed moderate to severe (based on qSOFA) COVID-19 | Standard of Care (81) versus  early steroid group (132) | Standard of care 64,  Early steroid 61 | Standard of care 50.6%  Early steroid 51.5% | Admitted to intensive care unit:  26.8% | 8 |
| **Fang Mei Yang**  **40** | China, Anhui | Provincial Hospital | 8 April 2020 | 22 January to 1 March, 2020 | Retrospective observational | 78 | Until viral clearance | Hospitalized COVID-19 patients | Non severe with steroid (9) versus  Non severe without steroid (46).  Severe with steroid (16) versus  Severe without steroid (7)  Severe patient median SOFA-score of 2 at first day of admission | Non severe with steroids mean age 40.2  Non severe without steroids mean age 39.9  Severe with steroids mean age 60.6  Severe without steroids mean age 54.3 | Non severe with steroids 55.6%  Non severe without steroids 47.8%  Severe with steroids 75%  Severe without steroids 71.4% | Severe: 29.5% | 8 |
| **Feng Ling Bai**  **66** | China, Wuhan, Shanghai,  Anhui | Jinyintan Hospital, Shanghai Public Health Clinical Center, Tongling People's Hospital | 10 April 2020 | 1 January to 15 February, 2020 | Retrospective observational | 476 | NR | Hospitalized COVID-19 patients | Moderate (352), severe (54), critical (70)  Moderate (Fever, cough, and other  symptoms)  Severe (respiratory distress, SaO2 <  <93% or P/F <300  mm Hg)  Critical type (  mechanical ventilation/shock occurs/organ dysfunction/  requiring ICU monitoring) | Moderate median 51  Severe median 58  Critical median 61 | Moderate 54.0%  Severe 61.1%  Critical 68.6% | Severe: 9.5%  Critical: 14.7% | 8 |
| **Fernandez Cruz et al.**  **41** | Spain, Madrid | Hospital Puerta de Hierro-Majadahonda | 22 June, 2020 | 4 March to 7 April, 2020 | Retrospective observational | 463 | NR | Adult patients with COVID-19 pneumonia complicated with ARDS and/or an hyperinflammatory syndrome | Steroids (396) versus  controls (67) | Steroids mean 65.4,  Control mean 68.1 | Steroids 69.7,  Control 61.2 | Severe ARDS  Steroids: 7.1  Controls: 0 | 8 |
| **Gazaruzzo**  **42** | Italy | Hospital group San Donato | June 17, 2020 | NR | Retrospective | 219 | Not reported | patients admit-  ted to hospital for SARS-CoV2 | Hospitalized patients with  Steroids versus without steroids | 68.5 | 50% | NR | 7 |
| **Gong Guan Jin**  **43** | China, Hubei | Yi Chang Central People's Hospital | 22 May 2020 | 30 January to 20 February, 2020 | Retrospective observational | 34 | NR | Hospitalized COVID-19 patients under 50 years old | Steroid (18) versus non-steroid (16) | Steroids 38.22,  Non-steroids 33.75 | Steroids 61.1,  Non-steroids 68.8 | 47.1% severe patients | 8 |
| **Huang Song Xu**  **45** | China, Beijing | Chinese PLA General Hospital | August 1, 2020 | 13 January – 10 March 2020 | Retrospective | 64 | NR  Maximum days follow-up was 29 days. | All COVID-19 patients | High dose methylprednisolone versus low dose methylprednisolone | All patients 47.8 | 58% | 33% | 6 |
| **Horby et al**  **23** | United Kingdom | 179 NHS hospitals | 22 June 2020 | 19 March to 8 June, 2020 | Randomized controlled trial | 6425 | 28 days | Hospitalized COVID-19 patients | Steroid (2104) versus usual care (4321) | Steroids 66.9,  Usual care 65.8 | Steroid s 64 %,  Usual care 64% | Invasive mechanical ventilation 15.6% | NA |
| **Hu wang hu**  **44** | China, Wuhan | Wuhan pulmonary hospital | July 11, 2020 | January 14 – February 9, 2020 | Retrospective | 308 | Until March 14, 2020 | COVID-19 pneumonia admitted to the  hospital | Glucocorticoid therapy versus non-glucocorticoid therapy | Glucocorticoid group median age = 54  Non-glucocorticoid group  median age = 48 | Glucocorticoid group 47.2%  Non-glucocorticoid group 46.7% | 0% | 8 |
| **Huang Yang Sang**  **81** | China, Jiangsu provence | 12 hospitals | May 26, 2020 | January 24 – April 20 2020 | Retrospective | 60 | Until April 20 | Severe cases of covid | Clinical characteristics in the improvement and deteri-  oration subgroups. | 57 | 58.3% | 13.3% | 6 |
| **Jeronimo**  **36** | Brazil | Tertairy care facility Manaus |  | April 18 – June 16 | RCT | 393 | Discharge or death | Clinical and/or radiological suspicion of COVID-19 | Methylprednisolone(MP) versus placebo | MP mean age 54  Placebo mean age 57 | MP 64.9%  Placebo 64.3% |  | RCT |
| **Keller**  **73** | USA , New York | Montefiore Medical Center | July 22, 2020 | March 11 – April 13, 2020 | Retrospective | 1806 | Until death or discharge | Patients admitted with a positive COVID-19 who either died or  had been discharged from the hospital. | Early glucocorticoids versus no glucocorticoids | Early glucocorticoids 61.7  No glucocorticoids 62.3 | Early glucocorticoids 50.7%  No glucocorticoids 53.7% |  | 8 |
| **Li Hu Song**  **46** | China | First College of Clinical Medicine Science, China Three  Gorges University, Yichang, | NR | NR | NR | 206 | NR | patients with COVID-19 | High dose methylprednisolone versus low dose methylprednisolone | No data available | No data available | NR | 4 |
| **Li Li Yin**  **47** | China, Shanghai | Shanghai Public Health Clinical Center | July 7, 2020 | 20 January – 10 June 2020 | Retrospective | 475 | Until July 6, 2020  (all patients had been dis-  charged or died during the hospitalization) | Adult patient with non-severe COVID-19 | Corticosteroid versus non-corticosteroid group | Corticosteroid 59  Non-corticosteroid 50 | Corticosteroid 60%  Non-corticosteroid 53.6% | NR | 8 |
| **Li Zhou li**  **48** | China, Shanghai | Multicentre | August 5, 2020 | January 20 – February 20 | Retrospective | 187 | 20 days | COVID-19 patients showing marked radiographic progression. | Early start group versus control group | Early start group mean age 57.6  Control group mean age 59.0 | Early start group 57.1%  Control 63.8% | 9% | 8 |
| **Lui Fang**  **Deng**  **49** | China, Hubei provence | nine tertiary hospitals in Hubei  province | May 5, 2020 | December 30, 2019 – January 24, 2020 | Retrospective | 137 | NR | Patients tested  positive for the nucleic acids of this CoV were identified as confirmed cases and enrolled in the study. | Clinical characteristics | 55 | 44.5% | 11.7% | 6 |
| **Liu Zheng Huang 50** | China, Zhuhai | Fifth Affiliated Hospital of Sun Yat-sen University | 29 May 2020 | 22 January to 2 March, 2020 | Retrospective observational | 101 | NR | Consecutive hospitalized COVID-19 patients | Non-severe with steroid (0), non-severe with non-steroid (75).  Severe with steroid (15) versus severe with non-steroid (9) | Non-severe mean age 44.0  Severe mean age 56.8 | Non-severe 38.7%  Severe 57.7% | 25.7% severe and critical patients | 5 |
| **Liu Zhang Wu**  **82** | China, Wuhan | Wuhan infectious disease hospital | July 31, 2020 | December 29 – February 28 2020 | Retrospective | 1190 | March 2  Endpoint in-hospitall deterioration and/or death | Patients  with at least two consecutive times of positive results  from high-throughput sequencing or real-time reversetranscriptase polymerase chain reaction (RT-PCR) assay  of nasal and pharyngeal swab specimens were confirmed  with COVID-19 | Corticosteroids versus no corticosteroids | All patients median age 57 | All patients 53.4% | 3.5% | 6 |
| **Lu Chen Wang**  **51** | China, Wuhan | Tongji Hospital | 19 May 2020 | 25 January to 25 February, 2020 | Retrospective observational and case-control analysis | 244 | NR | Hospitalized adult COVID-19 patients | Cohort study:  Steroid (151) versus non-steroid (93)  Case control: steroid (31) versus non-steroid (31) | Steroid 64  Non steroid 59 | Steroid 55%  Non steroid 48% | All patients were critically ill | 8 |
| **Ma Qi Deng**  **52** | China | Multicenter | August 1, 2020 | Janaury – March 2020 | Retrospective | 72 | Until discharge, max 40 days | Severe and critical COVID-19 | Corticosteroid versus non-corticosteroid | Corticosteroid 60  non corticosteroid 61 | Corticosteroid 60% | 20% versus 6% | 8 |
| **Ma Zeng Zhan**  **53** | China, Hunan | Multicentre | August 12, 2020 | January 23 – March 8 2020 | Retrospective | 450 | Maximumfollow-up29 days | Symptomatic COVID-19 patients | Corticosteroids versus no corticosteroids in severe and non-severe cases | Non-severe cortico+ 49.5  Non-severe cortico- 41.0  Severe cortico+ 54.5  Severe cortico - 64.5 | Non-severe cortico+ 53.1%  Non-severe cortico- 48.0%  Severe cortico+ 58.1%  Severe cortico - 60.0% | 43% | 8 |
| **Majmundar**  **54** | USA, New York City | Metropolitan Hospital Center | September 9, 2020 | March 16 – April 30, 2020 | Retrospective | 205 | Until May 10 | patients admitted to the non-ICU wards with laboratory-confirmed COVID-19 pneumonia | With or without corticosteroids | Corticosteroid mean age 58.67  Without corticosteroids mean age 57.18 | Corticosteroid 86.67%  Without corticosteroids 69.67% | 32.7% | 8 |
| **Mikulska**  **55** | Italy, Genova | San Martino University Hospital | August, 20 2020 | NR | Retrospective | 215 | Max. FU-days 56, median 49 days | Adult patients with COVID-19 pneumonia, not intubated, not treated with remdesivir and not pregnant. | Patients treated with standard of care (SOC) versus SOC with tocilizumab and/or methylprednisolone after 3 days of hospitalization versus SOC with tocilizumab and/or methylprednisolone within 3 days from hospitalization | 67.5 versus 65.9 (methylprednisolone versus tocilizumab) | 71.1% versus 82.8% | 24.9% | 8 |
| **Nelson**  **56** | USA, New York City | Large academic hospital and smaller community hospital New York | August 9, 2020 | March 1 - April 12, 2020 | Retrospective | 117 | 60 days | Patients with COVID-19 who required mechanical ventilation | Methylprednisolone versus control | Methylprednisolone median age 60  Control group median age63 | Meyhylprednisolone 67%  Control group 70% | 100% | 8 |
| **Rodriquez-Bano**  **57** | Spain | Multicentre | August 9, 2020 | February 2 – March 31 2020 | Retrospective | 1014 | 21 days | Adult patients from the COVID19Spain cohort | No treatment versus intermediate high dose corticosteroids (IHDC) versus pulse dose corticosteroids(PDC) versus combination tocilizumab/corticosteroids | IHDC median age 71  PDC median age 71  No treatment median age 69  combination group median age 65 | 72% IHDC  73% PDC  73% No treatment  72% combination | NR | 8 |
| **Rubio**  **68** | Spain | Hospital universitario Clinico san cecilio | 10 July 2020 | median follow-up 11 days | Retrospective | 92 | 11 days | Patients in the internal and intensive  medicine departments admitted for SARS-CoV-2 infection  confirmed by PCR and who met cytokine-release syndrome criteria | Glucocorticoids versus tocilizumab versus combination | All patients 63.9 (32-88) | All patients 63% | NR | 6 |
| **Salton**  **58** | Italy | 14 hospitals |  | February 27 – April 24 2020 | multicenter, observational, longitudinal study t | 173 | Until May 21 | SARS-CoV-2 positive, 2)  age >18 years and <80 years; 3) PaO2:FiO2 <250 mmHg; 4) bilateral infiltrates; 5) CRP >100 mg/L;  and/or 6) diagnosis of acute respiratory distress syndrome (ARDS) | early prolonged  methylprednisolone (MP) versus controls | MP mean age 64.4  Control group mean age 67.1 | MP 65.1%  Control 73.3% | 18.1% | 8 |
| **Shen Zheng Sun**  **59** | China, Shanghai | Shanghai Public Health Clinical Center | 23 Jun 2020 | 20 Jan to 29 Feb, 2020 | Retrospective observational | 325 | 28 days | Hospitalized COVID-19 patients | Glucocorticoids (27) versus no glucocorticoids (272) | 51 (in total cohort) | 51.7 (in total cohort) | Severe: 3.1  Critical: 4.9 | 6 |
| **Shi Wu Wang**  **71** | China, Zhejiang | First Affiliated Hospital of Zhejiang University | 2 Jul 2020 | 19 Jan to 17 Feb, 2020 | Retrospective observational | 99 | 28 days | Hospitalized COVID-19 patients | Virus negative (61) and virus positive (38) | Virus negative median age 50,  Virus positive median age 61.5 | Virus negative 57.4%  Virus positive 68.4% | NR | 6 |
| **Tomazini**  **37** | Brazil | Multicentre | September 2, 2020 | June 17 – June 23, 2020 | RCT | 299 | 28 days | Patients were at least 18 years old, had confirmed or suspected COVID-19 infection  and were receiving mechanical ventilation  within 48 hours of meeting criteria for moderate to severe  ARDS with partial pressure of arterial blood oxygen to frac-  tion of inspired oxygen (PaO2:FIO2) ratio of 200 or less. | Control versus dexamethasone 20mg/10mg | Dexamethasone mean age 60.1  Control mean age 62.7 | Dexamethasone 59.6%  Control 65.6% | NR | RCT |
| **Wang Yang Li**  **67** | China, Wuhan | Multicentre | March 16, 2020 | January 16 – January 29, 2020 | Retrospective | 69 | Until February 4 | NR | Clinical features | All patients median age 42 | All patients 46% | NR | 6 |
| **Wang Zhang Yu 69** | China | Tongji Hospital | April 23, 2020 | NR | Retrospective | 548 | 15 days | NR | Survivors versus non-survivors | NR | NR | 23% | 5 |
| **Wang Jiang He**  **60** | China, Wuhan | Union Hospital of Huazhong University of Science and Technology | 28 April 2020 | 20 January to 25 February, 2020 | Retrospective observational | 46 | Until discharge/death | Severe patients with COVID-19 pneumonia at the isolation ward | Steroid (26) versus non-steroid (20) | Steroids median age 54  Non-steroids median age 53 | Steroids 62%  Non-steroids 50% | All patients were severely ill | 8 |
| **Wu Chen Cai**  **61** | China, Wuhan | Jinyintan Hospital | 13 March 2020 | 25 December, 2019 - 6 January, 2020 | Retrospective observational | 201 | 18 - 50 days | Hospitalized COVID-19 patients | ARDS (84) versus non-ARDS (117) and ARDS survivors (27) versus ARDS non-survivors (23) | All patients median age 51 | All patients 63.7 % | Patients with ARDS according to WHO guidelines: 41.8% | 6 |
| **Wu Huang Zhu**  **62** | China, Wuhan | 2 centers | September 3, 2020 | December 26, 2019 – March 15, 2020 | Retrospective | 1763 | final follow-up date was March 19 | COVID-  19 hospitalized severe or critical cases | non-corticosteroid group versus systemic corticosteroid | Severe cases 63  Critical cases 68 | Severe cases 54.8%    Critical cases 59.1% | 13.9% | 8 |
| **Xu Chen Yuan**  **63** | China | First Affiliated Hospital, School of Medicine,  Zhejiang University and the Shenzhen Third People’s Hospital | April 9, 2020 | January 9, - February 19 2020 | Retrospective | 113 | 21 days | Patient admitted with (1) disease duration over 21 days without viral RNA  clearance, (2) viral RNA clearance occurred within 21 days, or  (3) death occurred within 21 days. | Viral shedding < or > 15 days with or without corticosteroids | All patients median age 52 (43-63)  Viral shedding duration < 15 days median age 48.0,  Viral shedding duration >15 day median age 54.4 | All patients 58.4%  Viral shedding duration < 15 days 40.5%  Viral shedding duration > 15 days 67.1% | 20.4% | 4 |
| **Yang Lipes**  **64** | Canada, Montreal | Jewish General Hospital | 11 May 2020 | NR | Retrospective observational | 15 | NR | COVID-19 patients admitted to ICU who received corticosteroids in the context of cytokine release syndrome | No group division | All patients median age 72 | All patients 60% | All patients were critically ill | 7 |
| **Zha Li Pan**  **65** | China, Anhui | Second People’s  Hospital of Wuhu and Yijishan Hospital in Wuhu, | 8 April 2020 | 24 January to 24 February, 2020 | Retrospective observational | 31 | NR | Hospitalized COVID-19 patients | Steroid (11) versus non-steroid (20) | Steroid media age 53,  Non-steroid median age 37 | steroid 73%  Non-steroid 60% | NR | 8 |

**Footnotes:** unless otherwise specified, severity was defined according to National Health Commission of the People's Republic of China guidelines, meaning that severe cases were defined as either respiratory distress (≥30 breaths/ min), oxygen saturation ≤93% in rest, or arterial partial pressure of oxygen (PaO2)/fraction of inspired oxygen (FiO2) ≤300 mmHg (1 mmHg=0.133kPa). Critical cases were defined as either respiratory failure and requiring mechanical ventilation, shock, or with other organ failure that required ICU care. **a)** defined according to WHO guidelines. **b**) according to guidelines of the American Thoracic Society and Infectious Disease Society of America.
Unless otherwise specified, time to viral clearance and viral shedding duration are defined as time to negative RT-PCR.

**+)** defined as not meeting the following criteria: (i) obvious alleviation of respiratory symptoms (eg. cough, chest distress and breath shortness) after treatment; (ii) maintenance of normal body temperature for ≥3 days without the use of corticosteroid or antipyretics; (iii) improvement in radiological abnormalities on chest CT or X-ray after treatment; (iv) a hospital stay of ≤10 days.

NOS score was based on **1)** mortality, **2)** viral clearance, or **3)** secondary outcomes.
