# Supplementary material for: Corticosteroid use in COVID-19 patients: a systematic review and meta-analysis on clinical outcomes
Source: Crit Care. 2020 Dec 14;24:696. doi: 10.1186/s13054-020-03400-9 (PMC7735177; doi:10.1186/s13054-020-03400-9)
Supplement: Supplementary file 6 — Additional file 6. Data extraction Treatment. [file 13054_2020_3400_MOESM6_ESM.docx]

**Supplement 6. Data extraction Treatment related variables**

| **Author** | **Reference** | **Type of steroid** | **Patients treated (number, %)**  **Describe subsets** | **Indication** | **Dose (mg/d)** | **Initiation of treatment (days) relative to start of symptoms/hospital admission** (has to be specified ) | **Duration of treatment (days)** |
| --- | --- | --- | --- | --- | --- | --- | --- |
| **Angus REMAP-CAP** | **34** | Hydrocortisone | Fixed 7 days 137 (34%) Shock 141 (35%)  Plabebo 101 (25%) | Randomization: admission ICU for respiratory or cardiovascular organ support | 200 mg/day Hydrocortison  ED 50 mg/day (prednisone) | Within 36 hours after ICU admission | Fixed 7 days  Shock: Treatment when Shock clinically evident |
| **Bani-Sadr** | **39** | Prenisone or Methylprednisolone | Before: 85  After: 172 | Adult Covid-19 pneumonia | 1 mg/kg Predisone | Hospital admission | 3-4 weeks |
| **Cao et al.** | **80** | Methylprednisolone | Survivors: 47.1  Non-survivors: 64.7  p=0.184 | NR | NR | NR | NR |
| **Chen Zhu Hong** | **70** | NR | 10.9% 29/267 (10.9) | NR | NR | NR | NR |
| **Chroboczek et al.** | **72** | NR | 21/70 (30) | NR | NR | From symptom onset, mean: 13.0±4.2 days | NR |
| **Dequin (CAPE COVID)** | **35** | Prednisone or methylprednisone | 76/149 (51 %) | Randomization: admission ICU or MC with respiratory failure, oxygen > 6 l/min | Hydrocortisone 200 mg/d or 7 days. (ED 50mg)  Then tapered to 100 (ED 25) mg/d 4 days, 50 (ED 12.5) mg/d 3 days.  Prednisone 50 mg/d, then tapered 25 mg/d 4 days, 25 mg/d 3 days.  When good response was present adjusted smaller dose schedule | Within 24 hours after ICU admission | 14 days |
| **Fadel et al.** | **38** | Methylprednisolone | Overall: 136 (63.89)  Standard of care: 46 (56.8)  Early steroid:  90 (68.2)  p=0.094 | Requiring ≥4 liters of oxygen per minute on admission, escalating oxygen requirements from baseline, or ICU-admission | 0.5-1 mg/kg/day in 2 divided doses  (ED 1.6 – 1.3mg) | Median time from admission:  Standard of care: 5 (3-7)  Early steroid: 2 (1-3)  p<0.001 | 3 to 5 days  For patients who required ICU admission: 3 to 7 days |
| **Fang Mei Yang** | **40** | Severe patients: IV methylprednisolone | General group: (9/55) 16.36  Severe patients: (16/23) 69.57 | According to severity and the individual opinion of clinicians. | Severe: 40 mg/day  (ED 50mg) | NR | Severe: 4.5 (3.0, 5.8) |
| **Feng Ling Bai** | **66** | NR | Overall: 127/476 (26.7%)  Moderate:  47/352 (13.4%)  Severe: 28/54 (51.9%)  Critical: 52/70 (74.3%)  p<0.001 | NR | NR | NR | NR |
| **Fernandez-Cruz et al.** | **41** | Methylprednisolone | Overall: 396/463 (85.5) | At the discretion of the treating physician | 1 mg/kg/day (or equivalent) or pulses  (ED 1.3mg) | Median time from symptom onset:  10 (8-13) days | NR  Patients treated with pulses received a median of 3 pulses (2-4) |
| **Gazzaruso** | **42** | Methylprednisolone  Prednison | 6, 2.7%  2, 0.9% | 2 for rheumatoid arthritis, 4 for chronic obstructive pulmonary disease,  1 for asthma, and 1 for inflammatory bowel disease | 6 patients used 5-12.5mg prednisone  2 patients used 4mg methylprednisolone (ED 5mg) | patients  were already taking corticosteroids  before the admission | NR |
| **Gong Guan Jin** | **43** | Methylprednisolone | 18, 52.9 % | Persistent high fever (body temperature> 39°) for three consecutive days, or progress in CT imaging within 2 days, or with hypoxia and difficulty breathing | 1-2 mg/kg/d (ED 1.3 -2.5)  gradually halved every 3 days | NR | 5 to 10 days |
| **Horby et al.** | **23** | Dexamethasone | 1975, 95%  Another 337, 7% in usual care group received steroids | Randomization: | 6 mg once daily  (ED 40mg) | Median time from symptom onset to corticosteroid treatment allocation:  8 (5-13) | For up to 10 days or until discharge if sooner |
| **Huang Song Xu** | **45** | methylprednisolone | 11 low-dose vs. 10 high dose | NR | <1.5mg/kg/day (mean 0.9mg/kg/day)  ED <1.9mg/kg/day, mean 1.1mg/kg  >1.5mg/kg/day (mean 3.3mg/kg/day)  ED >1.9mg/kg/day, mean 4.1mg/kg/day | NR | 6.8 days (low-dose) versus  11.6 days (high-dose) |
| **Hu wang hu** | **44** | Methylprednisolone  Prednisolon | 84, 28,2%  15, 4,9% | NR | glucocorticoid total equivalents (mg), median 200mg | Onset illness and glucocorticoid initiation 7 days | Median duration 6 days |
| **Jeronimo** | **36** | Methylprednisolone | 194, 49.8% | Randomization: SaO2 < 94% , extra need for oxygen or IMV | 0.5mg/kg 2dd  ED 0.6mg/kg 2dd | NR | 5 days |
| **Keller** | **73** | NR | 140, 7.75% | NR | NR | <48 hours of admission | NR |
| **Li Hu Song** | **46** | Methylprednisolone | NR | NR | High dose 80mg/day  ED 100mg/day  Low dose 40mg/day  ED 50mg/day | NR | NR |
| **Li Li Yin** | **47** | Prednisone  Methylprednisolone | 55, 11.6% | NR | 20mg/day (ED 2mg) n=5  40mg/day(ED 50mg)n=13  40mg/day(ED 50mg)n=17  20ng/day (ED 25mg)n=9  20mg/day(ED 25mg)n=11 | Within a median of 2 days on hospital admission (IQR 1-5 days) | 3 days  5 days  3 days  5 days  3 days |
| **Li Zhou li** | **48** | Methylprednisolone | 47, 25,1% | NR | 40-80mg/day  followed by 20mg/day  ED 50-100mg/day followed by 25mg/day | Patients were con-  sidered eligible for corticosteroids once chest  radiology examinations suggested they were at risk of  progression to ARDS. | 3 days  Total length treatment <7days |
| **Liu Zheng Huang** | **50** | Methylprednisolone | 15 patients, all severe  14,9 % of total population.  57.7% of total severe patients | One of the following: (1) PaO2/FiO2 ratio ≤ 150 mmHg, (2) persistent respiratory distress after high flow nasal oxygen therapy (duration ≥ 2 hours, gas flow ≥ 50L/min, FiO2 ≥ 0.6), or (3) multi-lobar infiltrates involving both lungs and an increase by ≥ 50% within 24-48 hours. | 2-8 mg/kg (ED 2.5-10mg) per day in general, no more than 500mg (ED 625mg) per day | NR | Pulse single-dosage: if patients met administration criteria again, the pulse was repeated |
| **Lui Fang Deng** | **49** | Methylprednisolone | 40, 29.2% | Patients who suffered from persistent high fever that did not subside or showed significant short-term disease progression determined by imaging results. | 30-80mg/day  (ED 37.5-100mg/day) | NR | 3–5 days |
| **Liu Zhang Wu** | **82** | NR | 289, 25.7% | NR | NR | NR | NR |
| **Lu Chen Wang** | **51** | Methylprednisolone, hydrocortisone or dexamethasone | 151, 61.9% | NR | Median prednison-equivalent dosage 50 (25–200) mg/day | NR | Median: 8 (4-12) |
| **Ma Qi Deng** | **52** | Methylprednisolone | 47, 65.3% | NR | 40mg/day (ED 50mg) n=42  80mg/day (ED 100mg) n=5 | NR | 3 days  3 days |
| **Ma Zeng Zhan** | **53** | Methylprednisolone | 126, 28% | NR | median daily doses 56.6mg (ED 70.8mg) | NR | Median time 5.0 (3.0-7.0) |
| **Majmundar** | **54** | Prednisolon, prednisone, dexamethasone, methylprednisolone | 60, 29.7% | NR | 80mg/day methylprednisolone  ED 100mg/day | Corticosteroid was commenced at a median of 2 days  following admission | Median 5 days |
| **Mikulska** | **55** | Methylprednisolone | Total 101, 51.5%  Methylprednisolone, 45, 23%  Methylprednisolone combined with tocilizumab, 56, 28.6%   \|  \| \| --- \| | NR | 1mg/kg (ED 1.3mg/kg)  0.5mg/kg (ED 0.6mg/kg) | NR | First 5 days  After 5 days, for 5 days |
| **Nelson** | **56** | Methylprednisolone | 48, 41% | NR | 1mg/kg (max 80mg)  (ED 1.3mg/kg met max 100mg) | At least 5-7 days after symptom onset and only in those with evidence of systemic inflammation | 5 days |
| **Rodriquez-bano** | **57** | Methylprednisolone | intermediate-high dose of corticosteroids (IHDC) 117, 15%    Pulse dose corticosteroids (PDC) 78, 10%  Combination tocilizumab/corticosteroids 151, 19% | Patients were assigned to tocilizumab, IHDC or PDC if administered  in 2 days after day 0 | PDC if 250 mg of methyl-  prednisolone or equivalent per day, or otherwise IHDC  (PDC ED 312.5mg) | NR | NR |
| **Rubio** | **68** | glucocorti-  coids | Glucocorticosteroids alone 60, 65.2%  Glucocorticosteroids combined with tocilizumab 23, 25% | NR | 2mg/kg/dag (n=30)  250mg/dag (n=27)  500mg/dag (n=26) | NR | 3 days |
| **Salton** | **58** | Methylprednisolone | 83, 48% | NR | 80mg/day (ED 100mg) | NR | At least 8days |
| **Shen Zheng Sun** | **59** | Methylprednisolone | Total: 15.4 | NR | NR | NR | NR |
| **Shi Wu Wang** | **71** | NR | Virus positive: 78.9  Virus negative: 77 | NR | Median initial dosage:  Virus positive: 60 mg/d (40-80)  Virus negative: 40 mg/d (40-80)  p=0.47 | Median time from symptom onset:  Virus positive:7.5 (5-10)  Virus negative: 8 (6-10)  p=0.35 | NR |
| **Tomazini** | **37** | Dexamethasone | 155, 52% | Randomization: intubation and need for mechanical ventilation , moderate to severe ARDS per Berlin, onset of ARDS before randomization < 48h | 20mg and 10mg  ED 133.3mg and 66.7mg |  | First 5 days 20mg, followed bij 10mg for 5 days |
| **Wang Jiang He** | **60** | Methylprednisolone | 26, 56.% | Severe COVID-19 pneumonia | 1-2 mg/kg/d (ED1.3 – 2.5mg) | Early administration, but NR | 5-7 days |
| **Wang Yang Li** | **67** | NR | 10, 14.5% | NR | NR | NR | NR |
| **Wang Zhang Yu** | **69** | NR | 341, 62% | NR | Median dose  survivors 422mg  non-survivors 300mg | NR | Median duration  survivors 111 days  non-survivors 4 days |
| **Wu Chen Cai** | **61** | Methylprednisolone | Without ARDS: 10.3  With ARDS: 59.5  p<0.001 | NR | NR | NR | NR |
| **Wu Huang Zu** | **62** | Methylprednisolone | critical cases 159, 63.8%  severe cases 532, 35.1% | NR | 40mg (ED 50mg) | Median initial time  of corticosteroid use since being diagnosed as critical cases  was 0.1 hours.  Median initial time of corticosteroid use since being diagnosed as severe cases was  2.2 hours. | 6 days in severe cases  5 days in critical cases |
| **Xu Chen Yuan** | **63** | Methylprednisolone | 64, 56.6 | NR | 0.5-1 mg/kg body weight (ED 0.6 – 1.3mg) | NR | NR |
| **Yang Lipes** | **64** | Methylprednisolone (9), hydrocortisone (4), dexamethasone (2) | 15, 100% | Hypoxic respiratory failure,  vasoplegic shock on multiple vasopressors or both respiratory and cardiovascular failure. | Median prednisolone equivalent dose over 24h: 200 (104-200) mg. | Median time from symptom onset: 14 (12–15) | NR |
| **Zha Li Pan** | **65** | Methylprednisolone | 35.5 | NR | 40 mg once or twice a day | Within 24 hours of hospital admission | Median: 5 (4.5-5.0) |

**Footnotes Table 2**: unless otherwise specified, severity was defined according to National Health Commission of the People's Republic of China guidelines, meaning that severe cases were defined as either respiratory distress (≧30 breaths/ min), oxygen saturation ≤93% in rest, or arterial partial pressure of oxygen (PaO2)/fraction of inspired oxygen (FiO2)≦300 mmHg (1 mmHg=0.133kPa). Critical cases were defined as either respiratory failure and requiring mechanical ventilation, shock, or with other organ failure that required ICU care. **a** = severity defined according to WHO guidelines. **+** = defined as not meeting the following criteria: (i) obvious alleviation of respiratory symptoms (eg. cough, chest distress and breath shortness) after treatment; (ii) maintenance of normal body temperature for ≥3 days without the use of corticosteroid or antipyretics; (iii) improvement in radiological abnormalities on chest CT or X-ray after treatment; (iv) a hospital stay of ≤10 days.
