# Supplementary material for: Corticosteroid use in COVID-19 patients: a systematic review and meta-analysis on clinical outcomes
Source: Crit Care. 2020 Dec 14;24:696. doi: 10.1186/s13054-020-03400-9 (PMC7735177; doi:10.1186/s13054-020-03400-9)
Supplement: Supplementary file 7 — Additional file 7 . Data extraction Outcome. [file 13054_2020_3400_MOESM7_ESM.docx]

**Supplement 7. Data Extraction Outcomes.**

| **Author** | **Mortality Definition**  (short-term, e.g 28-day, 30-day, in-hospital, overall) | **Mortality (%)**  **(quantification)** | ***Effect on Mortality***  ***(summary described in words)*** | **Viral Clearance**  **Definition**  (i.e. according to RT-PCR in respiratory specimens) | **Viral clearance (days)**  **(quantification)** | ***Effect on viral clearance***  ***(summary described in words)*** | ***Effect on Length of hospital stay (days)*** | ***Effect on need for mechanical ventilation (%)*** | **Oxygenation Definition**  (as defined by the investigators) | ***Effect on oxygenation (quantification)*** | ***Effect on respiratory secondary outcomes (time on ventilator/ventilator free days)*** | ***Effect on secondary infection*** |
| --- | --- | --- | --- | --- | --- | --- | --- | --- | --- | --- | --- | --- |
| **Angus REMAP-CAP**  **ref 34** | In-hospital Mortality | Fixed 7 day course of hydrocortison 30% (41/137)  Shock-dependent course of hydrocortison  26% (37/141)  Placebo 33% (33/99) | Relative to the no steroids group, the median adjusted odds ratios from the primary model were (fixed dose) 1.03 (95 CI 0.53-1.95), (shock dose) 1.10 (95 CI 0.58-2.11) yielding 54% and 62% Bayesan posterior probabilities of superiority. | NR | NR | NR | Mean adjusted hazard ratio length of stay hospital  Fixed 0.99  Shock 0.94 Probability of superiority of hydrocortison  Fixed 43%  Shock 31% | NR | NR | NR | Mean adjusted odds ratio Respiratory support free days  Fixed 1.45  Shock 1.31  Probability of hydrocortison superiority Fixed 94%  Shock 85% | NR |
| **Bani-Sadr**  **ref 39** | Death within 16 days median | Before-periode(Corticosteroids not recommended): n = 17 (20%)  After-period (use of corticosteroid therapy): n = 31 (18%) | The after period was not associated with a lower risk of death (HR 0.86 95% CI 0.47 – 1.56). | NR | NR | NR | NR | NR | The after period (use of corticosteroids) more frequent oxygen therapy was required). | Oxygen use Before  n = 52 (61,9%)  After  n = 125 (76,7%)  Maximal oxygen therapy  Before 5 litres  After 5,7 litres | NR | Antibiotic therapy  Before 80 (95%)  After 162 (95,9%)  (broad spectrum antibiotics) |
| **Cao et al.**  **ref 80** | Overall mortality | Overall mortality 16,7%  Died in corticosteroid group n=11/51 (64.7%) p=0.184 | No differences in mortality among those who did or did not receive steroids. | NR | NR | NR | NR | NR | NR | NR | NR | NR |
| **Chen Zhu Hong**  **ref 70** | NR | NR | NR | A cycle threshold (Ct)  value of 40 or more was defined as negative in at least two sequential respiratory  tract samples collected at more than a 24 -h interval | Median duration of viral  shedding since illness onset  18.0 days corticosteroids vs. 12.0 days no corticosteroids  P<0.001  Hazard ratio (95% CI) 0.60 (0.39–0.94) P=0.02  4 | Corticosteroid use was  associated with delayed clearance of viral RNA | NR | NR | NR | NR | NR | NR |
| **Chroboczek et al.**  **ref 72** | NR | NR | NR | NR | NR | NR | NR | NR | NR | NR | Corticosteroid therapy  affected the risk of intubation with a risk difference (ATE) of -47.1% (95% CI -71.8 to -22.5)  Odds ratio (95% CI) < 0.001 (3.24 x 10-06 to .09) p=.004 | NR |
| **Dequin (CAPE COVID)**  **ref 35** | Death on day 21 | Steroid n=11 (14,7%)  Placebo n= 20 (27,4) | The observed post hoc analysis of proportions of death at day 21 was not statistically significant.  Study underpowered due to stop of the trial. | NR | NR | NR | NR | Day 21 Steroid: n=17 on mechanical ventilation(22.7 %)  Placebo:n= 17 on mechanical ventilation (23,3 %) | Day 21  High flow oxygen therapy versus  Low flow oxygen therapy | High flow  Steroids n=3 (4%)  Placebo:n=0 0%)  Low Flow Oxygen Steroids n=1 (1,3%)  Placebo n=4 (5,5 %) | Prone position  Steroid: 36 (47%)  Placebo:39 (53,4%)  ECMO  Steroid 2 (2,7%)  Placebo:2 (2,7%)  Inhaled nitric oxide(iNO):  steroids 5 (6.7%)  placebo 11 (15%) | infections on day 28  Steroids:n=28 (37,7%)  Placebo:n=30 (41%) |
| **Fadel et al**  **ref 38** | In-hospital all-cause mortality. | Standard of care: n=21, 26.3%  Early steroid: n=18, 13.6%  OR 0.45 (95% CI 0.22-0.91)  p=0.024 | methylprednisolone had a reduced rate of the pri-  mary composite endpoint of death, ICU transfer, and mechan-  ical ventilation | NR | NR | NR | median hospital length of stay in days  Standard of care: n=8 (5-14)  Early steroid: n=5 (3-7)  p<0.001 | Overall:  Standard of care:n= 36 (44.4%)  Early steroid: n=38 (28.8%)  p=0.020  Respiratory failure requiring mechanical ventilation:  Standard of Care: n=26 (36.6%)  Early steroid: n=26 (21.7%)  p=0.025 | NR | NR | NR | NR |
| **Fang Mei Yang**  **ref 40** | NR | NR | NR | ∼2–3 throat swabs or sputum  samples were routinely collected once per week from all patients  for reverse transcription-polymerase chain reaction (RT-PCR) test-  ing to assess viral clearance. If the RT-PCR test result was nega-  tive, the test was repeated the next day to avoid false-negative re-  sults. | Time to SARS-CoV-2 RNA clearance (days),  mean ± SD  Severe patients:  Steroid: 18.8±5.3  Non-steroid: 18.3±4.2 | there was no significant differ-  ence identified in both patients in the general group  and patients in the severe group | NR | NR | NR | NR | NR | NR |
| **Feng Ling Bai**  **ref 66** | Overall mortality | death in critical patients n=21/52 (40.4%) | NR | NR | NR | NR | Hospital length of stay - days  Steroids: 22 days (17-32)  Non-steroid: 15 days (11-22)  p<0.001 | NR | NR | NR | NR | NR |
| **Fernandez Cruz**  **ref 41** | In-hospital mortality | Steroids: n=16, 13.9%  Controls: n=55, 23.9%  HR 0.36 (95% CI 0.14-0.93)  p=0.035 | In-hospital mortality was lower in patients treated with steroids than in controls.  Steroid treatment reduced mortality by 41.8% relative to no steroid  treatment (relative risk reduction, 0.42 [95% confidence interval, 0.048 to 0.65]). | NR | NR | NR | NR | NR | PaO2/FiO2 (mmHg) | Steroids: 263 (112.1)  Controls: 267 (78.9)  p=0.878 | NR | NR |
| **Gazzaruso**  **ref 42** | NR | 0% vs. 36.5% in ‘without steroids’ | NR | NR | NR | NR | NR | NR | NR | Oxygen saturation 90.7% versus 88.0% (no corticosteroids) | NR | NR |
| **Gong Guan Jin**  **ref 43** | NR | NR | NR | The time  of viral nucleic acid negative conversion is calculated as the time  interval between the two consecutive negative detections of new  coronavirus nucleic acid and the onset of illness | Steroid: 29.11±6.61  Non-steroid: 24.44±5.21  p<0.05 | Time required for nucleic acid  negative conversion in the corticosteroids treatment group was  longer than that in the non‐corticosteroids treatment group | NR | NR | P/F ratio  mmHg | Steroid: 236.61 ± 47.13  Non-steroid:358.19 ± 38.82 | Corticosteroids therapy can ef-  fectively improve oxygenationand prevent disease progression. | NR |
| **Horby**  **ref 23** | 28-day mortality | Dexamethasone: 22.9%  Usual care: 25.7%  (rate ratio, 0.83; 95% con-  fidence interval [CI], 0.75 to 0.93; P<0.001) | Mortality at 28 days was significantly lower in  the dexamethasone group than in the usual care group | NR | NR | NR | Median duration of hospitalization  Dexamethasone: 12 days  Usual care: 13 days | Dexamethasone: 5.7%  Usual care: 7.8% | NR | NR | The risk of progression to invasive mechanical  ventilation was lower in the dexamethasone  group than in the usual care group (risk ratio,  0.77; 95% CI, 0.62 to 0.95) | NR |
| **Huang Song Xu**  **ref 45** | Death rate | n= 0/11,0% low dose versus n=2/10, 20% high dose | There were no significant differences in death rate | NR | NR | NR | the duration of hospitalization  was significant longer in the high-dose group  mean  23.3 days (low-dose) versus 32.3days (high-dose)  p=0.01 | NR | NR | NR | Respiratory support  mean  13.3 days (low-dose) vs. 11.6 days (high-dose).  Not significant | NR |
| **Huang Yang Shang**  **ref 81** | NR | 0% | There were  no statistically significant differences between the 2  groups in  administration of  glucocorticoid | NR | NR | NR | NR | NR | NR | NR | NR | Four of 34 patients developed  secondary infections, but there were no statistically sig-  nificant differences between the improvement and deteri-  oration subgroups. |
| **Hu wang hu**  **ref 44** | Mortality | 0% | NR | A cycle threshold value (Ct-value)  of 40 or more for the two genes was defined as negative. | Median Days  glucocorticoid group: 18 days (15−23) versus non-glucocorticoid group 20 days (18−23)  p= 0.55 | There was no significant difference with regard to the negative  conversion rate of viral nucleic acid and duration time of negative  transformation of viral nucleic acid between the glucocorticoid therapy  and the non-glucocorticoid therapy groups. | NR | NR | NR | NR | There was no statistical difference in  the cure rate of COVID-19 pneumonia patients between the gluco-  corticoid therapy and non-glucocorticoid therapy groups (73 [84.9 %]  vs 15 [83.3 %], p = 0.85). | NR |
| **Jeronimo**  **ref 36** | 28-day mortality | Steroids: 37.1% versus 38.2% placebo  P=0.629 | no evidence of improved survival in the overall population with a short course of  intravenous methylprednisolone.  However, a subgroup analysis found a lower  mortality in patients over 60 years who received methylprednisolone. | NR | NR | NR | median days:  methylprednisolone 10 days  versus  9 days placebo  p=0.296 | Need for mechanical ventilation  MP 34%  versus  placebo 33.7% | NR | NR | No significant difference was seen between patients. | Sepsis was not higher in patient using methylprednisolone |
| **Keller**  **ref 73** | in-hospital mortality | Odds radtio 1.13 (0.71-1.80) | Early treatment with glu-  corticoids is not associated with decreased mortality. | NR | NR | NR | NR | Mechanical ventilation  Odds ratio 1.55 (0.88-2.73) | NR | NR | Early treatment with glu-  corticoids is not associated with decreased need for mechanical ventilation . | NR |
| **Li Hu Song**  **ref 46** | NR | NR | NR | No definition  suggestion from manuscript is that HR eludes to risk of no more viral shedding after 30 days | high-dose (80 mg/day; aHR, 0.67 [95%  CI, .46–.96]; P = .031) versus low-dose  corticosteroids (40 mg/day; aHR, 0.72  [95% CI, .48–1.08]; P = .11) | high-dose (but not low-dose)  corticosteroids potentially  delayed viral shedding of patients with  COVID-19. | NR | NR | NR | NR | NR | NR |
| **Li Li Yin**  **ref 47** | In-hospital-mortality | Corticosteroids n=1/55, 1.8%.  Non corticosteroids 0/55, 0%. | There was no significant difference between the two groups in mortality   \|  \| \| --- \| | Two consecutive negative tests for SARS-CoV-2 with at least 24-h intervals. | Median days  Corticosteroids 18 days versus non-corticosteroids 11 days.  P<0.001 | Prolonged virus clearance time in steroid group | Corticosteroid 23 days (17–28) versus non-corticosteroids 15 days (12–20) | NR | NR | NR | NR | Corticosteroids increased the use of antibiotics.  corticosteroids = 49 (89.1%) versus non-corticosteroids 13 (23.6%)  p=0.001 |
| **Li Zhou li**  **ref 48** | NR | NR | NR | NR | NR | NR | NR | Mechanical ventilation early-start group 10.6% [5/47]) versus control group 33.3%  [7/21]  P = 0.037 | NR | NR | The proportion of patients requiring invasive mechan-  ical ventilation was significantly lower in the early-start group than in the control group.  The duration from onset of symptoms to invasive mechanical ventilation did not significantly differ  between two groups. | NR |
| **Liu Zheng Huang**  **ref 50** | NR | NR | NR | SARS-CoV-2 negative-conversion days of each patient | Steroid: 10.0 ± 5.3 days  Non-steroid: 10.0 ± 7.9 days | no statis-  tical difference | NR | NR | NR | Oxygenation improved significantly, no numbers reported | NR | NR |
| **Lui Fang Deng**  **ref 49** | NR | NR | Treatment neither significantly  shortened the disease course nor improved the prognosis. | NR | NR | NR | NR | NR | NR | NR | NR | NR |
| **Liu Zhang Wu**  **ref 82** | In-hospital death | Glucocorticoids group n=76/289, 59.8% | Administration  of glucocorticoids seemed to increase the risk of deterioration to severe disease after admission. | NR | NR | NR | NR | NR | NR | NR | NR | NR |
| **Lu Chen Wang**  **ref 51** | 28-day mortality | Steroid: n=79/151, 52.3%  Non-steroid: n=5/93, 5.4%  (adjusted OR 1.05; 95% CI 0.15–7.46)  p>0.3 | Every 10-mg increase in dosage was associated with additional 4% mortality risk  (adjusted HR 1.04, 95% CI 1.01–1.07) p=0.003 | NR | NR | NR | NR | NR | SpO2/FiO2 (mmHg) | SpO2/FiO2:  Steroid: 259 (121-303)  Non-steroid: 297 (279-388)  p<0.01  Case-control:  SpO2/FiO2:  Steroid: 291 (212–452)  Non-steroid: 294 (246–396)  p=0.57 | NR | NR |
| **Ma Qi Deng**  **ref 52** | Non-survivor | Corticosteroid group n=2/47, 4.3% versus non-corticosteroid group n=2/25, 8.0% | Corticosteroid therapy could not reduce the mortality | SARS-CoV-2 RNA RT-PCR tests revealed negative twice | Mean corticosteroid group 16.1 days versus non-corticosteroid group 19.4 days.  p=0.184 | Not associated with delayed viral clearance.  Time of viral clearance seemed to decrease in corticosteroid group. | Mean corticosteroid group 18.7 days versus non-corticosteroid group 21.0 days.  p=0.212 | NR | NR | Mean time of mechanical ventilation corticosteroid group 9.6days versus non-corticosteroid group 12.8 days.  p=0.376 | NR | NR |
| **Ma Zeng Zhan**  **ref 53** | Mortality (not further specified) | n=3/62, 4.8% in severe cases with corticosteroids  n=0/64, 0% in non-severe cases with corticosteroids | it was not  possible to assess the association between corticosteroid use and  mortality due to:  1) low death rate among the study  cohor;  2) the study was designed and aimed to describe the use of  corticosteroids among COVID-19 patients rather than assessing the  association between corticosteroid use and mortality outcome. | viral shedding was defined as the time from the date of  symptom onset to the date when two consecutive throat-swab with an  interval more than 24 h were negative for viral species. | In severe cases: median 19.0 (cortico +) versus 17.5 days  p=0.123  in non-severe cases  median 20.0 (cortico +) versus 17.0 days  p=0.001 | results demonstrated a significant  prolonged viral shedding time existed in nonsevere patients  receiving corticosteroid treatment. | In severe cases: Median 20 (cortico+) versus 16 days  p=0.091  In non-severe cases, median  19 (cortio+) versus 12.0 days  P<0.001 | Need for mechanical ventilation  In severe cases: n=9/62, 14.5 % (cortico+) versus n=1/20, 5.0% (cortico-)  p=0.461 | NR | NR | NR | Antibiotics  in severe cases:  95.2%(cortico+) versus 60.0%  p<0.001  in non-severe cases:  79.7%(cortico+) versus 33.9% (cortico-)  p<0.001  The proportion of patients receiving antibiotic therapy  in the corticosteroid group was significantly higher than in the  noncorticosteroid group. |
| **Majmundar**  **ref 54** | in-hospital death | n=8/60, 14.5% (in steroid versus 34/145, 22% (in non-corticosteroids)  p=0.114 | Patients who received corticosteroids were  found to have a lower in-hospital death rate, however not statistical significance likely due to a smaller sample  size. | NR | NR | NR | Length of stay (days), median  9 days in corticosteroid-group versus 7 days in non-corticosteroids.  p=0.025 | intubated  18.9% in corticosteroid-group versus 25.35% in non-corticosteroids.  p=0.334 | SpO2/Fio2 ratio  Median | Steroids: 190 (IQR,  92.5–298.44) versus  Non-steroid: 339.29 (IQR, 278.13–419.05)  P < 0.001 | NR | NR |
| **Mikulska**  **ref 55** | Death during follow-up | methylprednisolone: n=13/45, 28.9%  Tocilizumab: n=4/29, 13.8%  Combined therapy n=5/56, 8.9% | Early tocilizumab/methylprednisolone improved overall survival | NR | NR | NR | NR | NR | PaO2/Fio2, median mmHg | Methylprednisolone 201 versus tocilizumab 203 | No difference | NR |
| **Nelson**  **ref 56** | 60-day mortality | Methyprednisolon n=15/48, 31%  versus  Control 29/69, 42%.  p=0.236 | A statistically nonsignificant trend toward reduced mortality in methylprednisolone group | NR | NR | NR | Hospital discharge day 28: steroids 17% versus control 19%.  p=.776  Hospital discharge day 60 steroids 45% versus control 36%.  p=.374 |  | NR | NR | Increased probability of extubation  28 day v entilator free-days (mean) 6.21 methylprednisolon versus 3.14 control.  p=0.058 | NR |
| **Rodirquez-Bano**  **ref 57** | 21-day mortality | Intermediate-high dose of corticosteroids(IHDC)  18.8% (22/117)  p=0.08(versus no treatment)  Pulse dose of corticosteroids(PDC) 10.3% (8/78)  p=0.84 (versus no treatment) | No significant  association between Intermediate-high dose of corticosteroids or combination therapy and outcome. | NR | NR | NR | NR | Mechanical ventilation  IHDC 1.7%(2/117)  PDC 5.1% (4/78)  no treatment 5.5% (19/344) | NR | Need for oxygen suppletion  IHDC 6.8% (8/117)  PDC 10.3%  (8/78)  No treatment 4.7%  (16/344) | NR | NR |
| **Rubio**  **ref 68** | In-hospital-mortality | Tocilizumab  11.1% (1/9)  Hazard ratio 1  glucocorticoid pulses  8.3% (5/60)  Hazard ratio 0.064 (0.03-1.181) p=0.065  Combination  4.4% (1/23)  Hazard ratio 0.02 (0.0004-0.835) p=0.040 | The combination of glucocorticosteroid pulses seems to be associated  with a better prognosis of the disease, with a clear tendency  towards lower mortality | NR | NR | NR | NR | Intubation  Tocilizumab  16.7% (1/6)  Glucocorticoid pulses  5% (3/60)  Combination 4.4% (1/23) | NR | NR | Lower need for intubation | NR |
| **Salton**  **ref 58** | 28-day mortality | MP-treated patients n=6/83, 7.2%  versus  Control-group 21/90, 23.3%  p=0.05 | MP-treated patients had a 28 day lower risk of all-cause death than untreated ones | NR | NR | NR | The hospital length of stay did not differ between the groups | Mechanical ventilation  Methylprednisolone  n=14/83,16.9%  versus Control  26/289, 28.9%  p=0.095 | NR | NR | Significant increasement of mechanical ventilation-free  days (p=0.003) and median invasive mechanical ventilation days in methylprednisolone group (p=0.031) | NR |
| **Shen Zheng Sun**  **ref 59** | Case fatality rate | Severe patients:  Glucocorticoids: 13.04%  No glucocorticoids: 0%  p=0.681  All patients (mild+severe)  Glucocorticoids: 12%  No glucocorticoids: 0%  p<0.05 | As deaths only occurred in the severe-critical group, this comparison was inadequate to draw inferences on the impact of glucocorticoid use. | Time from onset to negative nucleic acid  detection in pharyngeal swab/blood sample | in median days.  Mild patients blood sample  Glucocorticoid: 18 days (11-22)  No glucocorticoid: 13 days (9-16)  p=0.003  Mild patients, pharyngeal swab  Glucocorticoid: 8 (6-11)  No glucocorticoid: 8 (5-11)  p=0.436  Severe-critical patients, blood sample:  Glucocorticoid: 22.5±8.7  No glucocorticoid: 25.7±7.5  p=0.502  Severe-critical patients, pharyngeal swab:  Glucocorticoid: 15.4±7.24  No glucocorticoid: 8.3±0.58  p=0.170 | Patients with mild disease using glucocorticoid tended to have longer viral shedding in blood | Median in total cohort: 15 days | In total cohort 7.1% | NR | NR | NR | NR |
| **Shi Wu Wang**  **ref 71** | NR | NR | NR | Shedding  cessation was defined as the occurrence of 2 consecutive RT-PCR  negative results of respiratory specimens in a 24-hour interval. | Unadjusted Hazard ratio 0.85 (95% CI 0.46-1.57)  P = 0.599  Adjusted Hazard ratio 1.00 (95% CI 0.53-1.89)  p=0.990 | Corticosteroid treatment was not independent factor of duration of SARS-CoV-2 RNA shedding | NR | NR | NR | NR | NR | NR |
| **Tomazini**  **ref 37** | All cause mortality 28-days | Dexamethason 85/151 (56.3%)  versus  Standard care 91/148 (61.5%)  Adjusted hazard ratio 0.97 (0.72 to 1.31) p= .85 | Mortality rates were high and not significantly different  between groups | NR | NR | NR | NR | NR | NR | Ventilator free days Dexamethasone 6.6 versus Standard care 4.0  p=0.04  Mechanical ventilation duration in days  dexamethason 12.5 versus standard care 13.9  p=0.11 | Intravenous dexamethasone plus standard care, com-  pared with standard care alone, resulted in a statistically sig-  nificant increase in the number of ventilator-free days  (days alive and free of mechanical ventilation) over 28 days. | Secondary infection  dexamethasone 33/151 (21.9%) versus standard care 4/148 (29.1%)  Absolute difference (95% CI)  7.2 (−3.3 to 17.7) |
| **Wang Jiang He**  **ref 60** | Mortality rate | Steroid: 2/26 (7.7%)  Non-steroid: 0/20 (0%)  p=0.714 | No  significant difference of mortality rate was observed between  the two groups (P = 0.714). | NR | NR | NR | NR | Steroid: 11.5% (3/26)  Non-steroid: 35% (7/20)  p=0.05 | NR | Steroid use resulted in faster improvement of SpO2, no numbers reported. | NR | NR |
| **Wang Yang Li**  **ref 67** | Mortality rate | Corticosteroids-treated group: n=4/10, 40%  Overall 5/67, 7.5% of total patients | Because of the limited sample in this work, the correlation be-  tween use of corticosteroids and death should be interpreted with  caution. | NR | NR | NR | NR | NR | NR | In corticosteroid group SpO2<90 6/10, 60%,  SpO2 >90 4/10, 40% | NR | NR |
| **Whang Zhang Yu**  **ref 69** | 15-day mortality | Total steroid group 19% (65/341)  Low Dose steroid  10.4% (26/249)  High dose Steroid  42.4%  (39/92(  Low dose versus high dose P<0.0001 | Low-dose glucocorticoid treatment or no glucocorticoid use was associated with a lower  hazard compared with high-dose treatment. | NR | NR | NR | NR | NR | NR | NR | NR | NR |
| **Wu Huang Zhu**  **ref 62** | 28-day mortality | In critical cases:  Corticosteroid group 44% (70/159)  versus non corticosteroid-group  15.6%(14/90)  p<0.001  In severe cases:  Corticosteroid group  15.9% (83/531)  versus  Non-corticosteroid group  2.6% (26/983)  P<0.001 | Compared with the  non-corticosteroid group, the in-hospital mortality was sig-  significantly higher in the corticosteroid use group. | NR | NR | NR | NR | NR | NR | NR | NR | NR |
| **Wu Chen Cai**  **ref 61** | NR | Methylprednisolon 46% (23/50)  Control  61.8% (21/34)  p=0.003  (Hazard ratio 0.38; 95% CI 0.20-0.72) | Among patients with ARDS, steroids decreased the risk of death compared to non-steroids. | NR | NR | NR | NR | NR | NR | NR | NR | NR |
| **Xu Chen Yuan**  **ref 63** | In-hospital mortality in 21 days | 1.8% (2/113) of all patients  Unknown how much in the corticosteroid group. | NR | viral RNA clearance in the first 21 days after ill-  ness onset.  Endpoint: 3 consecutive samples negative, with the first neg-  ative sample defining the duration of shedding).  a Ct value of 40 or more was defined as a negative result. | In corticosteroid group 40.5% (37/117)viral shedding <15 days  while  65.4% (76/117) viral shedding  ≥15 days | Corticosteroid (P = .025)  was  related to prolonged viral RNA shedding time.  OR 1.38, 95% CI .52–3.65 | NR | NR | NR | NR | NR | NR |
| **Yang Lipes**  **ref 64** | In-hospital mortality | Corticosteroid group: 20%, 3/15. | No comparison to a control group | NR | NR | NR | NR | NR | P/F ratio | An average increase in the arterial partial pressure of oxygen/fraction of inspired oxygen (i.e., P/F) ratio of 44 was detected 24 hours after steroid administration. | NR | NR |
| **Zha Li Pan**  **65** | NR | 0 patients died | NR | NR | In median days  Steroid: 15 days (14-16)  Non-steroid: 14 days (11-17)  p=0.87 | NR | In median days  Steroid: 20 days (18-21)  Non-steroid: 17 days (15.5-19.5)  p=0.14 | NR | NR | NR | NR | NR |
