# Supplementary material for: Corticosteroid use in COVID-19 patients: a systematic review and meta-analysis on clinical outcomes
Source: Crit Care. 2020 Dec 14;24:696. doi: 10.1186/s13054-020-03400-9 (PMC7735177; doi:10.1186/s13054-020-03400-9)
Supplement: Supplementary file 8 — Additional file 8. Viral Clearance time. [file 13054_2020_3400_MOESM8_ESM.docx]

**Supplement 8. Viral Clearance time.**

**Table. Viral Clearance Time**  (most frequently defined as two consecutive negative RT-PCR on nasopharyngeal swabs, or a cycle time value of 40 or more).

|  | Corticosteroids | | | Placebo | | |
| --- | --- | --- | --- | --- | --- | --- |
|  | MEAN/MEDIAN | SD/IQR | participants | MEAN/MEDIAN | SD/IQR | participants |
| Zha Li | 15.0 | 14-16 | 11 | 14.0 | 11-17 | 20 |
| Shen, et al. | 10.0 | 7-16 | 50 | 8.0 | 5-11 | 275 |
| Ma Zheng severe | 23.0 | 16-31 | 11 | 18.0 | 13-23 | 11 |
| Ma Zheng non severe | 20.0 | 16-25 | 55 | 17.0 | 13-22 | 55 |
| Ma Qi | 16.1 | 6.11 | 47 | 19.4 | 9.42 | 25 |
| Hu Wang | 18.0 | 15-23 | 86 | 20.0 | 18-23 | 18 |
| Gong Guan | 29.11 | 6.61 | 18 | 24.4 | 5.21 | 16 |
| Fang Mei *severe* | 18.8 | 4.2 | 7 | 18.3 | 5.3 | 16 |
| Fang Mei *non severe* | 17.6 | 4.9 | 46 | 18.7 | 7.7 | 9 |
| Chen Zhu | 13.0 | 0.0 | 29 | 12.0 | 0.0 | 238 |

**Li Hu et al.: potentially delayed viral shedding in CS group
Li LI et al: Significant delay in viral clearance in CS group**
**Liu Zheng et al: non-significant difference
Shi et al: in bloodsamples and swabs non-significant difference
Xu Chen et al: Prolonged virus shedding in CS group.**
