# Supplementary material for: Corticosteroid use in COVID-19 patients: a systematic review and meta-analysis on clinical outcomes
Source: Crit Care. 2020 Dec 14;24:696. doi: 10.1186/s13054-020-03400-9 (PMC7735177; doi:10.1186/s13054-020-03400-9)

**Supplement 9. Corticosteroids and Mechanical Ventilation**

**Table s9.**

| **Author** | **Corticosteroids** | **Control** |
| --- | --- | --- |
| **Requirement of mechanical ventilation** | | |
| Dequin | 8/16 (50%) | 12/16 (75%) |
| Fadel | 26/132 (21%) | 26/81 (36.6%) |
| Jeronimo | 18/93 (19.4%) | 16/95 (16.8%) |
| Li Zhou Li | 10.6% | 33.3% |
| Ma Zheng Zhan | 9/62 (14.5%) | 1/20 (5%) |
| Majmundar | 11/60 (18.97%) | 36/145 (23.35%) |
| Salton | 14/83 (16.9%) | 26/90 (28.9%) |
| Wang Jiang He | 3/26 (11.5%) | 7/20 (35%) |
| Horby | RR 0.76 (0.61 - 0.96)  (in favor of CS) | |
| Keller | OR 1.55 (0.88 - 2.73) a OR 1.34 (0.71 - 2.52) | |
| **Ventilator Free days** | | |
| Angus | Adjusted OR Fixed dose CS group: 1.45 (0.34) Shock dose CS group: 1.31 (0.30) (in favor of CS) | |
| Nelson | 6.21 (+/-7.45) | 3.14 (+/-7.45) |
| **Time on Mechanical Ventilator** | | |
| Ma Qi Deng | 9.6 (SD 6.36) | 12.8 (SD 6.4) |
| Tomazini | 12.5 (11.2-13.8) | 13.9 (12.7-15.1) |
| **Other definitions used** | | |
| Rodriquez Bano | Combined endpoint Requirement of mechanical ventilation and/or Death:  No separate numbers for mechanicalventilation | |
| Rubio | Combined endpoint Requirement of mechanical ventilation and/or Death:  No separate numbers for mechanicalventilation | |
| Shen Zheng Sun | Overall mechanical ventilation:  No separate numbers for subgroups | |

**Figure S6 Funnel Plot**


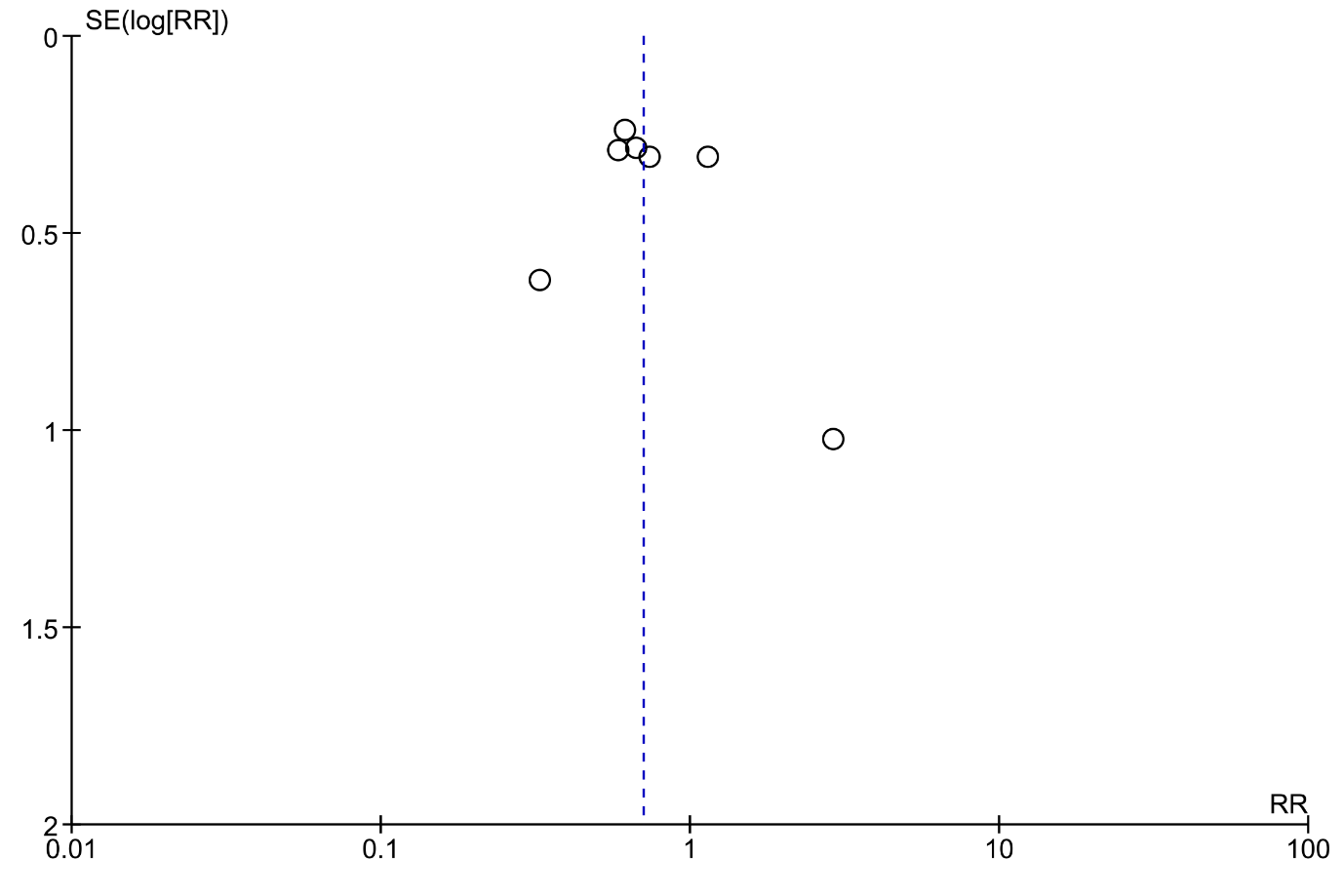

Supplement: Supplementary file 9 — Additional file 9. Mechanical ventilation. [file 13054_2020_3400_MOESM9_ESM.docx]
