# Supplementary material for: Corticosteroid use in COVID-19 patients: a systematic review and meta-analysis on clinical outcomes
Source: Crit Care. 2020 Dec 14;24:696. doi: 10.1186/s13054-020-03400-9 (PMC7735177; doi:10.1186/s13054-020-03400-9)
Supplement: Supplementary file 10 — Additional file 10. Grade classification. [file 13054_2020_3400_MOESM10_ESM.docx]

**Supplement 10. GRADE classification for main outcome measures.**

GRADE CLASSIFICATION for Mortality

Separately for RCTs and observational studies

| Study type | Risk of bias | Imprecision | Inconsistency | Indirectness | Publication bias | QUALITY of the EVIDENCE |
| --- | --- | --- | --- | --- | --- | --- |
| RCT’s | Not serious | Not serious ^a^ | Not serious | Not serious | Serious |   Moderate |
| Observational studies | Serious | Serious | Very serious | Not serious | Very serious |   Very low |

a: number of patients very large

GRADE CLASSIFICATION for Mechanical ventilation

All studies

| Study type | Risk of bias | Imprecision | Inconsistency | Indirectness | Publication bias | QUALITY of the EVIDENCE |
| --- | --- | --- | --- | --- | --- | --- |
| All studies | serious | serious | serious | Not serious | serious |   Very low |
