# Supplementary material for: Corticosteroid use in COVID-19 patients: a systematic review and meta-analysis on clinical outcomes
Source: Crit Care. 2020 Dec 14;24:696. doi: 10.1186/s13054-020-03400-9 (PMC7735177; doi:10.1186/s13054-020-03400-9)
Supplement: Supplementary file 11 — Additional file 11. Population bias. [file 13054_2020_3400_MOESM11_ESM.docx]

**Supplement 11. Origin study population for main outcome Mortality**

|  | Place | Hospital | Country | Inclusion period | Weight |
| --- | --- | --- | --- | --- | --- |
| Angus | - | Multicentre | Countries in  Europe, USA, Australia, New Zealand, Canada, | March 2020 – June 17, 2020 | 6.72 |
| Horby | - | 179 NHS hospitals | United Kingdom | March 19 – June 8, 2020 | 12.31 |
| Fadel | Michigan | Five hospitals in southeast and south-central Michigan | USA | March 12 – March 27, 2020 | 9.11 |
| Keller | New York | Montefiore Medical Center | USA | March 11 – April 13, 2020 | 3.44 |
| Majmundar | New York | Metropolitan Hospital Center | USA | March 16 – April 30, 2020 | 4.92 |
| Nelson | New York | Large academic hospital and smaller community hospital New York | USA | March 1 - April 12, 2020 | 1.00 |
| Dequin | Tours | Multi Center  Tours, Paris, Strasbourg, Limoges, Brest, Argenteuil | France | March 7 – June 1, 2020 | 6.72 |
| Bani Sadr | Reims France | University hospital of Reims | France | March 3 – April 14 2020 | 4.92 |
| Fernandez | Madrid | Hospital Puerta de Hierro-Majadahonda | Spain | March 4 – April 7, 2020 | 6.96 |
| Rodriquez | Sevilla | Multi Center  Sevilla, Barcelona, inst. Carlos III madrid, Hopital universitario La Paz, Hopital general universitario gregorio mranon Madrid | Spain | February 2 – March 31, 2020 | 5.09 |
| Li Li | Shanghai | Shanghai Public Health Clinical Center, admitted to the Infectious Diseases department. | China | January 20 – June 10, 2020 | 0.00 |
| Shen | Shanghai | Shanghai Public Health Clinical Center, visiting the Emergency Department | China | January 20 - February 29, 2020 | 0.00 |
| Cao | Wuhan | Zhongnan Hospital | China | January 3 – February 1, 2020 | 0.32 |
| Lu | Wuhan | Tongji Hospital | China | January 22 – February 25, 2020 | 0.09 |
| Wang | Wuhan | Union Hospital of Huazhong University of Science and Technology | China | January 20 – February 20, 2020 | 0.03 |
| Wu Huang | Wuhan | 2-centre  Hankou hospital, No. Six hospital | China | December 26, 2019 – March 15, 2020 | 2.19 |
| Wu Chen | Wuhan | Jinyintan Hospital | China | December 25, 2019 – January 6, 2020 | 9.41 |
| Ma Qi | Chongqing | Multi center  Chongqing public health medical center, Chongqing Three Gorges Central Hospital, Yongchuan Hospital of Chongqing Medical university | China | January – March 2020 | 0.00 |
| Mikulska | Genova | San Martino University Hospital | Italy | NR | 3.51 |
| Salton |  | Multi Center  Trieste, Memphis, Milano, Arezzo, Rome, Pravia, Milan, Udine | Italy/USA | February 27 – April 24 2020 | 8.54 |
| Jeronimo | Manaus | Tertairy care facility Manaus | Brazil | April 18 – June 16, 2020 | 5.78 |
| Tomazini | - | Multi center  Sao Paulo, Vila Velha, Rio de Janeiro, Sao Jose, Lisboa, Maringo, Sao Vicente de Paulo, Porte allegro, brasilia | Brazil | June 17 – June 23, 2020 | 8.73 |
